# Massive expansion and differential evolution of small heat shock proteins with wheat (*Triticum aestivum* L.) polyploidization

**DOI:** 10.1038/s41598-017-01857-3

**Published:** 2017-05-31

**Authors:** Xiaoming Wang, Ruochen Wang, Chuang Ma, Xue Shi, Zhenshan Liu, Zhonghua Wang, Qixin Sun, Jun Cao, Shengbao Xu

**Affiliations:** 1 State Key Laboratory of Crop Stress Biology for Arid Areas, College of Agronomy, Northwest A&F University, Yangling, Shaanxi 712100 China; 2 College of Life Sciences, Northwest A&F University, Shaanxi, 712100 China; 3 Department of Plant Genetics & Breeding, China Agricultural University, Yuanmingyuan Xi Road No. 2, Haidian District, Beijing, 100193 China; 4 Innovation Experimental College, Northwest A&F University, Shaanxi, 712100 China

## Abstract

Wheat (*Triticum aestivum*), one of the world’s most important crops, is facing unprecedented challenges due to global warming. To evaluate the gene resources for heat adaptation in hexaploid wheat, small heat shock proteins (sHSPs), the key plant heat protection genes, were comprehensively analysed in wheat and related species. We found that the sHSPs of hexaploid wheat were massively expanded in A and B subgenomes with intrachromosomal duplications during polyploidization. These expanded sHSPs were under similar purifying selection and kept the expressional patterns with the original copies. Generally, a strong purifying selection acted on the α-crystallin domain (ACD) and theoretically constrain conserved function. Meanwhile, weaker purifying selection and strong positive selection acted on the N-terminal region, which conferred sHSP flexibility, allowing adjustments to a wider range of substrates in response to genomic and environmental changes. Notably, in CI, CV, ER, MI and MII subfamilies, gene duplications, expression variations and functional divergence occurred before wheat polyploidization. Our results indicate the massive expansion of active sHSPs in hexaploid wheat may also provide more raw materials for evolving functional novelties and generating genetic diversity to face future global climate changes, and highlight the expansion of stress response genes with wheat polyploidization.

## Introduction

Diverse heat shock proteins (HSPs), one key component of cellular protein quality control network, are very important for plant heat stress adaptation^1–3^. Small heat shock proteins (sHSPs), one type of HSPs, are typically induced by heat, and function in protein folding and preventing or reversing substrate aggregation^3, 4^. SHSPs contain a conserved α-crystallin domain (ACD), preceded by a highly divergent N-terminal region and followed by a C-terminal extension. The ACD comprises seven or eight anti-parallel β-strands which form a β-sandwich. In their native state, sHSPs form oligomers (200–800 kDa) that operate as a functional unit^5, 6^. The ACD and C-terminal extension are the major regions responsible for oligomer formation, while the flexible N-terminal region is responsible for substrate recognition and oligomer stability^3, 4, 7–11^. Extensive studies have demonstrated that manipulating the sHSPs of plants remarkably altered their thermotolerance^12–16^, highlighting their role in heat adaptation. The composition and expression of sHSPs has recently been studied in several plants, including *Arabidopsis thaliana*, rice, algae, *Populus trichocarpa*, pepper, sugarcane and soybean, and the identified sHSPs have been categorized into sixteen subfamilies, including eleven cytoplasmic/nuclear localized (CI-CXI) and five organelle localized subfamilies^17–24^. However, the evolutionary mechanisms of sHSPs in plants, especially in plant polyploidization process, remain largely unknown.

Productivity of bread wheat (Triticum aestivum L.) cropping systems is at risk due to increasing temperatures as a result of global warming^25–27^. Bread wheat, also known as allohexaploid wheat (AABBDD), originated from two hybridization events among the genera *Triticum* and *Aegilops* ^28, 29^. The first hybridization, between *Triticum urartu* (AA) and a close relative *Aegilops speltoides* (BB), occurred <0.8 million years ago (Ma) and gave rise to the allotetraploid emmer wheat (*Triticum turgidum*; AABB). The second hybridization, between emmer wheat and *Aegilops tauschii* (DD), which arose from the hybridization of the AA and BB lineages ~5.5 Ma, occurred <0.4 Ma and gave rise to the allohexaploid wheat^30^. Owing to the high degree of autonomy in the subgenomes and the shorter evolutionary history^31^, bread wheat and its related progenitors provide a good system for studying the polyploidization process^30^.

Recent studies have suggested that all flowering plants have undergone at least two ancient polyploidy events during their evolutionary history^32^. The autopolyploid which resulted from whole genome duplication or unreduced gametes fusion within a single species, and the allopolyploidy which resulted from interspecific hybridisation, generate extra gene copies^33, 34^. These duplicated genes then underwent subfunctionalization, neofunctionalization, specialization, pseudogenization or concerted evolution, leading to greater genetic diversity^34–36^, which is the key factor in adaptation to new habitats and distribution to larger geographical areas than those covered by their progenitors^28, 34^. The genome size of hexaploid wheat approximates to the sum of three diploid progenitors with 2–10% DNA loss with polyploidization, suggesting that most genes are redundant compared with their progenitors and some genes may be lost^28^. As the key heat protection genes, twenty-seven candidate sHSPs have been identified in hexaploid wheat, with all found to localise to the mitochondria (26) or nucleus (1)^37^. This differed from the literature on the cellular localization of other identified sHSPs, which has shown that most sHSPs are actually located in the cytoplasm^17, 20, 22^. Further elucidation and clarification of sHSP candidates in wheat is therefore required.

With the accumulation of genome data on hexaploid wheat and its relatives, systematic analyses of sHSP evolution and function in Triticeae have become practicable. Here, we demonstrate the evolutionary pattern and mechanism of sHSPs in Triticeae and provide evidence for massive intrachromosomal gene duplications, selection pressure variations and the expression pattern diversity during wheat polyploidization. Our results provide fundamental evidence that will be valuable for further wheat sHSP applications as well as for its evolutionary pattern variations with polyploidization.

## Results

### Massive expansion of sHSPs during wheat polyploidization

**Figure 1:**
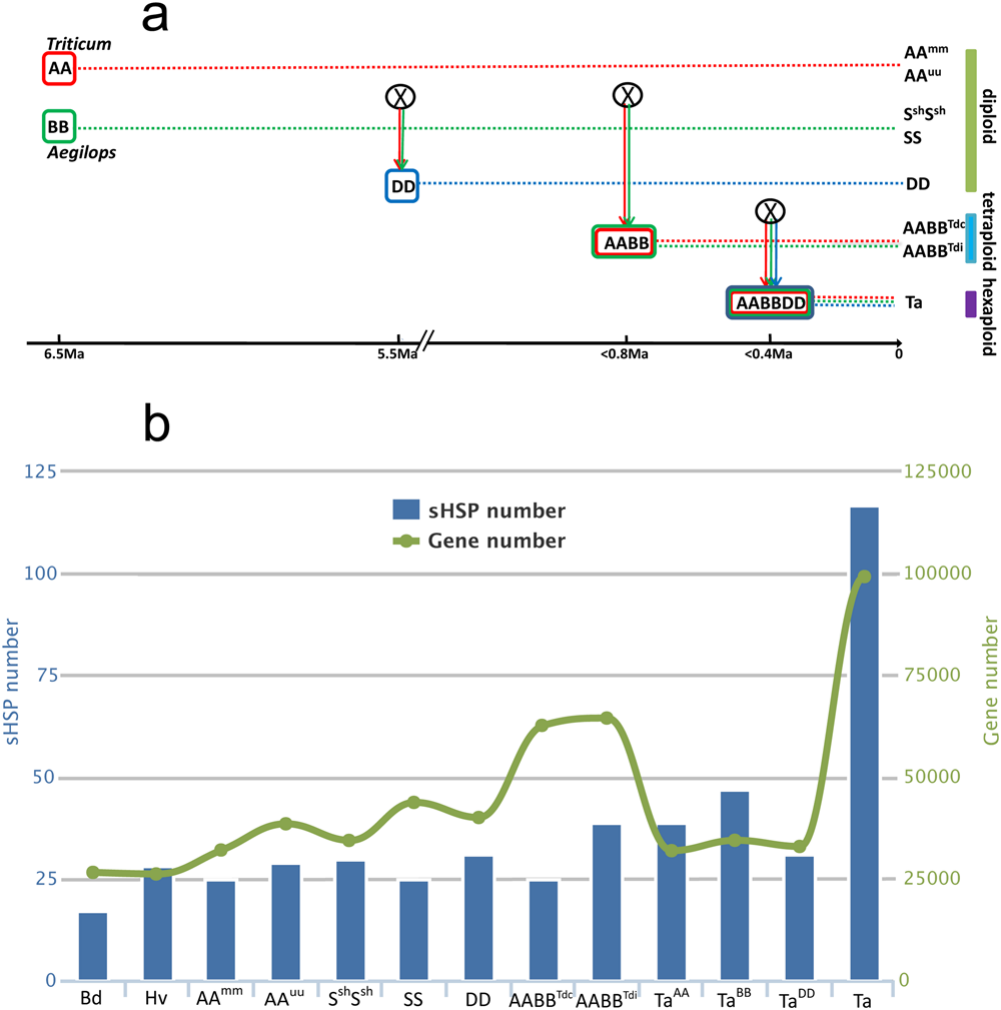
Number of sHSPs in wheat and related species. (**a**) Evolutionary history of bread wheat. The symbol ‘X’ in circle mark hybridisation event. This figure is modified from [Marcussen, T. *et al*. Ancient hybridizations among the ancestral genomes of bread wheat. *Science* 345 (2014)]^30^. Reprinted with permission from AAAS. (**b**) Numbers of sHSPs in different species. Left Y axis represents the identified sHSP numbers and right Y axis represents the gene numbers contained in the genome sequences analysed. Abbreviations: Ma, million years ago; Bd, *Brachypodium distachyon*; Hv, *Hordeum vulgare*; AA^mm^, *Triticum monococcum*; AA^uu^, *Triticum urartu*; S^sh^S^sh^, *Aegilops sharonensis*; SS, *Aegilops speltoides*; DD, *Aegilops tauschii*; AABB^Tdc^, *Triticum durum*, cv. Strongfield; AABB^Tdi^, *Triticum durum*, cv. Cappelli; Ta^AA^, subgenome A of bread wheat; Ta^BB^, subgenome B of bread wheat; Ta^DD^, subgenome D of bread wheat; Ta, *Triticum aestivum*.

To characterize the copy number variation of sHSPs during wheat polyploidization, we performed comparative analyses on hexaploid wheat and its relatives (diploid and tetraploid). The evolutionary relationships among Triticeae were constructed (Fig. 1a), with reference to the results of Marcussen *et al*.^30^. Diploid progenitors were represented by *T. urartu* (AA^uu^) and *Triticum monococcum* (AA^mm^) as the wheat subgenome A donors, *Ae. Tauschii* (DD) as the wheat subgenome D donor, and *Ae. sharonensis* (S^sh^S^sh^) and *Ae. speltoides* (SS) as the wheat subgenome B donors. Tetraploid progenitors were represented by *T. turgidum* (Cappelli, AABB^Tdc^) and *T. turgidum* (Strongfield, AABB^Tdi^) as the reference AABB genomes^29–31, 38–41^.

With reference to the sHSP identifications in *A. thaliana* and rice^17, 20, 22^, 404 sHSP candidates were identified in Triticeae, including 321 *sHSP* genes and 83 *Acd* genes (which shared homology with the ACD domain of sHSPs but were divergent from sHSPs), respectively (Supplementary Fig. S1). About 25 to 31 *sHSP* genes were identified in seven diploid and tetraploid relatives (Fig. 1b, Supplementary Table S1), suggesting that this stable amount of sHSPs may be sufficient to maintain the heat adaptation capability of Triticeae. Surprisingly, 117 *sHSPs* were identified in bread wheat, many more than in its relatives, even remarkably more than the total (56–70) of a tetraploid (AABB) and the DD progenitors, or the sum (81–90 in total from AA, BB and DD) of its three diploid progenitors (Fig. 1b, Supplementary Table S1). In a previous study, the copy number variation of wheat genes was analysed, which showed that the gene numbers of all six investigated gene families in hexaploid wheat were considerably less than the sum of the three diploid progenitors^31^. These results indicate that the sHSPs, as environmental adaptation genes, were unusual and remarkably expanded in hexaploid wheat with polyploidization.

Tetraploid wheat (AABB^Tdc^ and AABB^Tdi^) originated recently (around 0.8 Ma)^30^. Our result shows that the genome size of current tetraploid is close to the total of its two diploid progenitors, along with 13.1% (AA^uu^ and S^sh^S^sh^ to AABB^Tdi^)–31.2% (AA^uu^ and SS to AABB^Tdc^) gene loss (Fig. 1b). However, the number of sHSPs in tetraploid wheat was similar to that in its diploid progenitors, rather than reflecting the sum of the two diploid progenitors (Fig. 1b). In hexaploid wheat, the genome size approximates to the sum of the three diploid progenitors, along with 2–10% DNA loss^28^. The number of sHSPs in hexaploid wheat, however, was much higher than the sum of its three diploid progenitors (Fig. 1b). Further genomic comparison revealed remarkable sHSPs expansion in subgenomes A and B, but not in subgenome D (Fig. 1b, Supplementary Table S1), in consitent with that the genes in subgenome D keep relatively stable with polyploidization^42–44^.

### The sHSPs of hexaploid wheat expanded in the CI and CII subfamilies with intrachromosomal duplications

To further understand sHSP expansion during wheat polyploidization, the sHSPs of Triticeae were classified into 13 known subfamilies (Supplementary Fig. S1). No sHSPs belonging to the CIV, CVII and CXI subfamilies were detected among Triticeae. However, interestingly, our analysis identified a new subfamily specific to Triticeae that was designated as CXII onwards.

**Figure 2:**
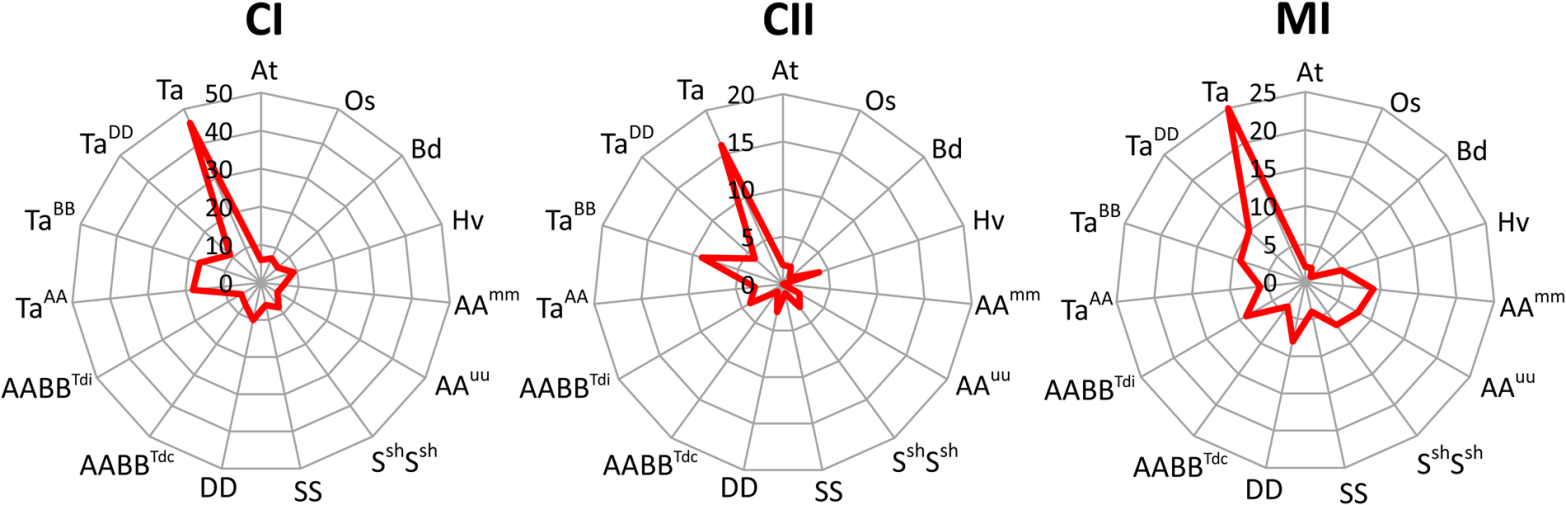
Copy number variation of CI, CII and MI subfamily during wheat polyploidization. Abbreviations, At, *Arabidopsis thaliana*; Os, *Oryza sativa*; Bd, *Brachypodium distachyon*; Hv, *Hordeum vulgare*; AA^mm^, *Triticum monococcum*; AA^uu^, *Triticum urartu*; S^sh^S^sh^, *Aegilops sharonensis*; SS, *Aegilops speltoides*; DD, *Aegilops tauschii*; AABB^Tdc^, *Triticum durum*, cv. Strongfield; AABB^Tdi^, *Triticum durum*, cv. Cappelli; Ta^AA^, subgenome A of bread wheat; Ta^BB^, subgenome B of bread wheat; Ta^DD^, subgenome D of bread wheat; Ta, *Triticum aestivum*.

The subfamilies CI, CII contained the most members among the 14 identified sHSP subfamilies in Triticeae. The sHSPs belonging to these two subfamilies also exclusively showed remarkable expansion in hexaploid wheat compared with the total number of sHSPs among the three diploid progenitors, or among the tetraploid and DD progenitors (Fig. 2, Supplementary Table S1). Gene expansion in subgenome A was mainly associated with the CI subfamily, while gene expansion in subgenome B was mainly associated with the CI, CII subfamilies.

**Figure 3:**
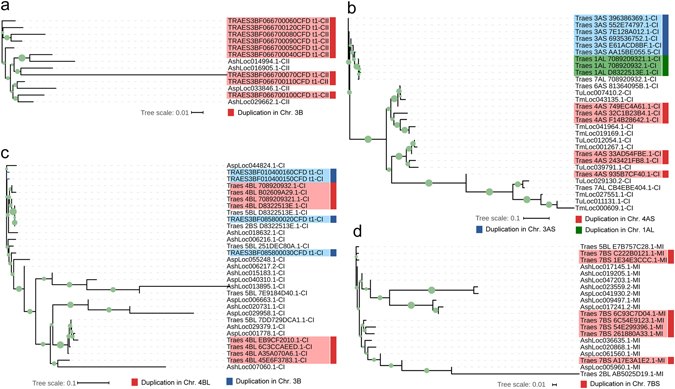
Gene expansions of subfamilies CI, CII and MI in subgenomes A and B. (**a**) Duplications of subfamily CII in subgenome B. (**b**) Duplications of subfamily CI in subgenome A. (**c**) Duplications of subfamily CI in subgenome B. (**d**) Duplications of subfamily MI in subgenome B. The bootstrap values are plotted as circles at the nodes with the circle size proportional to the bootstrap value. Abbreviations: Asp, *Aegilops speltoides*; Ash, *Aegilops sharonensis*; Tm, *Triticum monococcum*; Tu, *Triticum urartu*; Traes, *Triticum aestivum*.

Interestingly, these expanded sHSPs were enriched in specific chromosome fragments. For example, remarkable CII sHSP expansion occurred in subgenome B (Fig. 2, Supplementary Table S1), and all nine CII sHSPs in subgenome B were located in a 1 Mb region of chromosome 3B (Supplementary Fig. S2), suggesting the expanded sHSPs may be the result of intrachromosomal duplications. Further sequence comparative analysis demonstrated that these nine genes expanded from 1–3 original sHSPs during polyploidization (Fig. 3a). Similarly, sHSP expansion in the CI subfamily occurred particularly in chromosomes 4AS, 3AS, 1AL (Fig. 3b), 4BL and 3B (Fig. 3c), and the sHSPs of the MI subfamily mainly located in chromosome 7BS (Fig. 3d). These results suggest that sHSP expansion in hexaploid wheat originated from intrachromosomal duplications, which is consistent with the segmental duplications of sHSPs observed in *A. thaliana*, rice and soybean^22, 23, 45^, and exhibit a notably higher intrachromosomal duplication ratio during wheat polyploidization^31, 41^.

However, not all sHSP subfamilies displayed expansion in hexaploid wheat. Subfamilies CIII, CX and CXII exhibited decreased numbers of sHSPs compared with the diploid or tetraploid progenitors (Supplementary Fig. S3). This result suggested that sHSP loss should not be overlooked as it occurs simultaneously with gene expansion during wheat polyploidization.

### Severe purifying selection acts on sHSPs during wheat polyploidization

**Figure 4:**
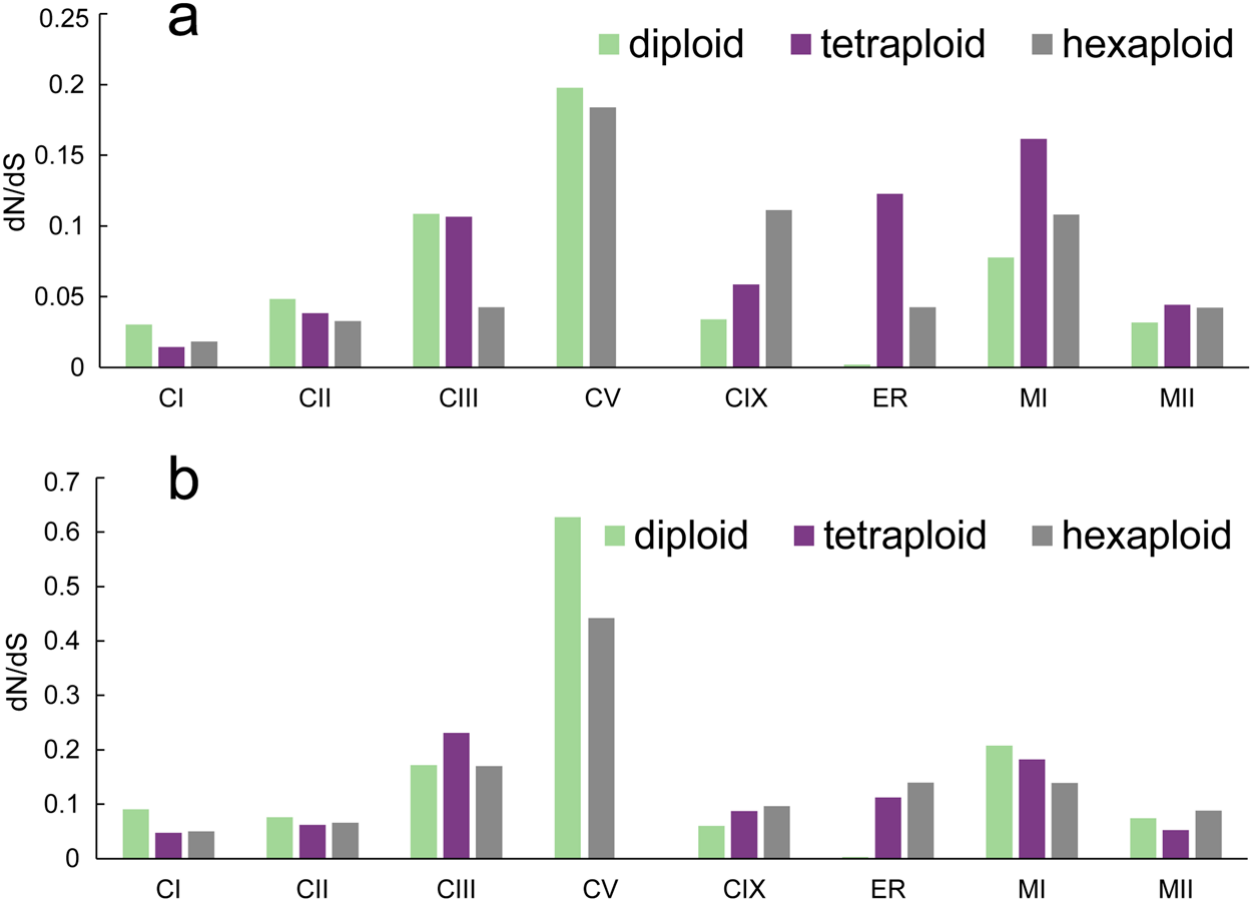
Selection pressure variation during wheat polyploidization. (**a**) The selection pressures acting on the ACD domain region. (**b**) The selection pressures acting on the full sHSPs. The X axis represents the different sHSP subfamilies and Y axis represents the dN/dS value.The dN/dS values were estimated based on the total sHSPs of diploid, tetraploid and hexaploid genomes, respectively. If the sHSP number was lower than three, the dN/dS value was not calculated.

Previous studies have demonstrated that different subfamilies of sHSPs possess distinct evolution histories in plants^4^. To unveil the underlying evolutionary mechanism of each sHSP subfamily in Triticeae and the natural selection acted on duplicated sHSPs in hexaploid wheat, we estimated the ratio (ω) of nonsynonymous (dN) to synonymous (dS) substitution rates (ω = dN/dS) with codon-based models provided in the PAML programs^46^. The ω values estimated by the one-ratio model were 0.05 and 0.1 for the ACD domain and the full length proteins, respectively, indicating that strong purifying selection acted on this protein family to remove nonsynonymous mutations and to maintain their important biological functions (Fig. 4). In general, different sHSP subfamilies were under different types of purifying selection, and the ω of each subfamily displayed similar purifying selection between the ACD domain and the full sHSP protein sequence, but weaker selection on the full sHSP sequence. This result is consistent with the fact that the conserved ACD domain is the core region for maintaining sHSP function.

The subfamilies CI, CII and P are the most ancient sHSPs that arose around 400 Ma, while other sHSPs evolved from these three subfamilies much more recently^47, 48^. Among the diploid progenitors, the ACD domain of the CI, CII and P subfamilies showed lower ω values (Fig. 4). In addition, the CIX and MII subfamilies also showed strong purifying selection compared with other subfamilies, such as the CIII, CV and MI subfamilies, which were under relatively weaker purifying selection. This finding was consistent with previous reports that CV sHSPs showed distinct expression patterns in *A. thaliana* and rice^20, 22^, indicating that they had experienced a function shift with weaker selection pressures.

In tetraploid relatives and hexaploid wheat, similar selection patterns were observed, indicating that these patterns were conserved across Triticeae relatives. For CI and CII subfamilies which experienced gene duplications, similar purifying selections were observed between hexaploid wheat and its progenitors, indicating that the gene duplications did not alter the selection pressures acted on these two subfamilies and the duplicated genes also under strong purifying selections. However, a stronger purifying selection was observed for the subfamily CIII in hexaploid wheat, while obvious weaker purifying selection acted on subfamilies of CIX and P (Fig. 4), suggesting that subfamilies CIII, CIX and P may experience altered selection pressure during the polyploidization process.

Taken together, these findings indicate that specific purifying selection is acting on each sHSP subfamily and is conserved across Triticeae relatives, which may lead different sHSP subfamilies in distinct evolutionary directions. Although massive gene expansions occurred, the sHSPs in hexaploid wheat have evolved in this manner, with the exception of minor differences in the CIII, CIX and P subfamilies.

### The sHSPs of hexaploid wheat were duplicated in divergent sHSP groups

Gene duplications, arising from single gene duplication or whole genome duplication events, result in enlarged and potentially more complex gene families. In this study, the sHSPs of the CI, CV, CIX, ER, MI and MII subfamilies could be divided into two groups evidently in the polygenetic tree, and these two groups were likely paralogs originating from gene duplications (Supplementary Fig. S4). These duplicated sHSPs showed obvious species preferences; for example, the groups CIb and ERb were only present in monocots, whereas the groups CIXb, MIb and MIIb were unique to Triticeae. These results indicated that the original duplications, which led to the different groups of sHSPs, were ancient events that mostly related to speciation, rather than to the wheat polyploidizaion.

To understand the evolutionary fates of duplicated genes, the codon-based models contained in PAML^46^ were employed (Supplementary Table S2). Analysis using the one-ratio model (model 0) and the two-ratio branch model (model 2) showed that the two groups of CIX, ER and MI subfamilies had different ω values, indicating asymmetrical evolution between the duplicated groups. The site-specific discrete model (model 3) and the clade model (model D) were applied to detect divergent selective pressure. The results demonstrated that the selective pressures that acted on the two groups of the CI, CV, ER, MI and MII subfamilies were distinctly different (Supplementary Table S2), thus suggesting that functional divergence occurred between paralogs. Furthermore, many type I sites (conserved in one duplicated cluster but highly variable in another duplicated cluster) and type II sites (highly conserved in two duplicated clusters but variable between them) responsible for functional divergence were identified (Supplementary Fig. S5). In general, the number of type II sites identified was greater than the number of type I sites (only type II sites were identified in the CV, ER and MII subfamilies), and these sites were mainly located in the ACD regions. This finding further supported the theory that the two groups of these subfamilies had experienced substantial functional divergence.

To test the effect of positive selection on functional divergence, the branch-site model A (model A) and the null model A were applied (Supplementary Table S2). Different positive selection sites were detected between the leading branches of CIa/CIb and ERa/ERb. However, two duplicated groups of CI and ER, displayed the same positive selection sites, suggesting that positive selection occurred before gene duplication and was not therefore the reason for functional divergence.

In conclusion, the duplicated clades of the CI, CV, ER, MI and MII subfamilies were under distinct selection pressure acting on the ACD domain, which led to functional divergence. However, such gene duplications and functional divergences were ancient events, occurring long before hexaploid wheat polyploidization. Furthermore, the expansion of sHSPs in hexaploid wheat occurred in both of the diverged groups, suggesting that the expansion of sHSPs was not related to the functional divergence of sHSPs, but simply to an increase in the numbers in the diverged sHSP group.

### Weaker purifying selection and evident positive selection in the N-terminal region conferred sHSPs with more flexibility

**Figure 5:**
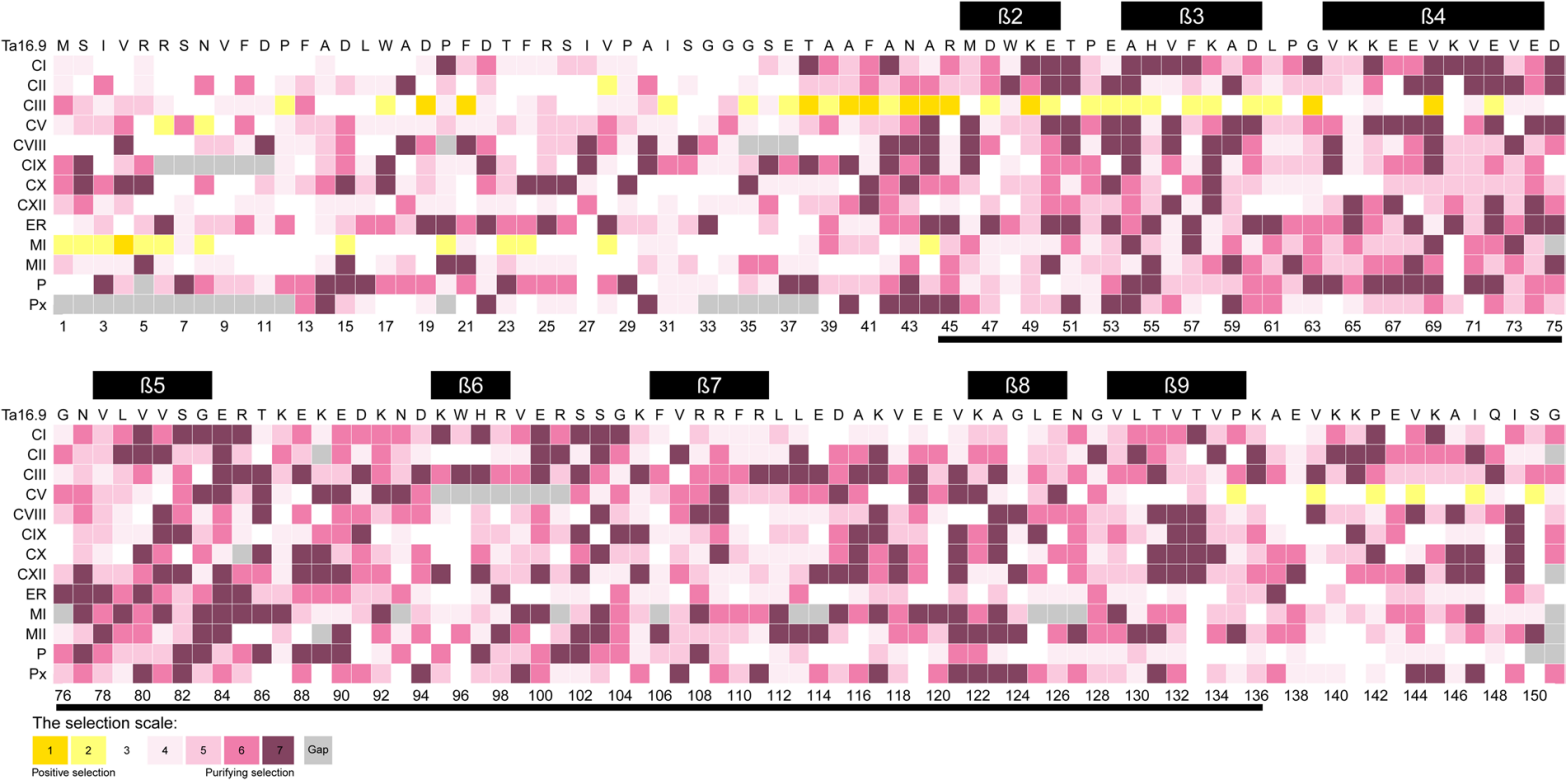
Selection pressures acting on each residue of the sHSPs. The protein sequences of each subfamily were aligned to the sHSP 16.9 of *Triticum aestivum* (Ta16.9), for which the crystal structure has been reported^6^, and selection pressures acting on each residue were estimated in each subfamily. The black line at the bottom marks the ACD domain. The protein sequence of Ta16.9 and its secondary structure (β-strands) is shown at the top. The selection scale is shown at the bottom.

To evaluate the selection pressures that acted on each region of the sHSPs in Triticeae, we aligned the sHSP sequences of each subfamily to Ta16.9, the model used for sHSP studies^6^, to detect the selection pressures that acted on each amino acid residue (Fig. 5). The results showed that no conserved residue was under strong purifying selection across all subfamilies, even in the β-strands, with the most conserved region being in the ACD^3^. Generally, the residues in the ACD and the C-terminal extension regions were under stronger purifying selection than those in the N-terminal region. By contrast, the positive selection sites identified were mainly located in the N-terminal region of the CIII (eight of 11 residues located in the N-terminal) and MI (one residue located in the N-terminal) subfamilies. The N-terminal region plays a major role in substrate recognition and binding^3, 4, 7–11^. Thus, the weaker purifying selection and the evident positive selection in the N-terminal region may confer greater flexibility in sHSPs, allowing the binding of more substrates.

### The sHSPs of hexaploid wheat are actively transcribed

**Figure 6:**
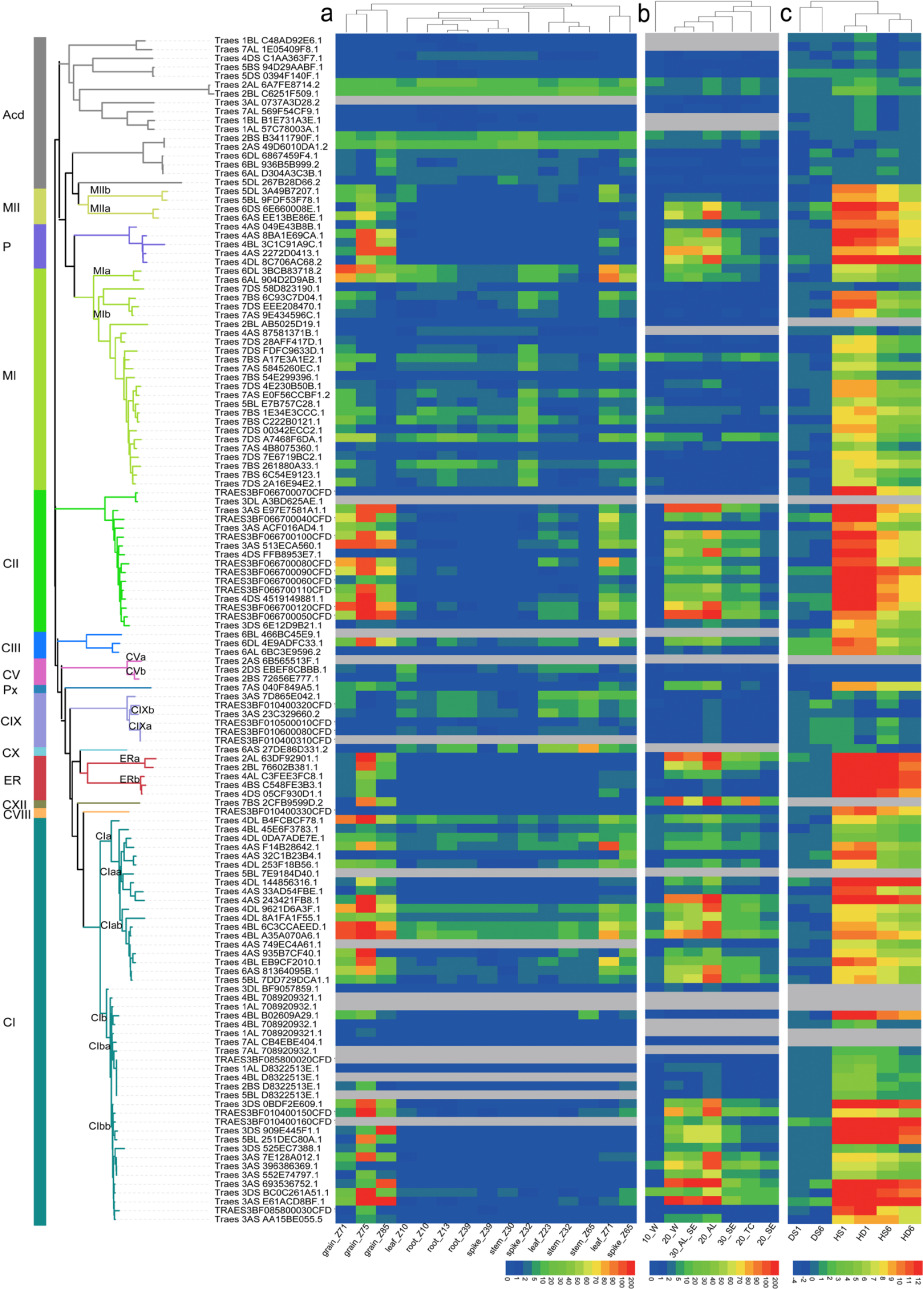
Phylogenetic tree of wheat *sHSP* genes and their expression profiles. The phylogenetic tree of wheat sHSP and Acd (that shared homology with the ACD domain of sHSPs but diverged from sHSPs) genes is shown on the left side of the panel. The clades of each subfamily are depicted by specific colours, and the names of subfamilies are marked to the left. The symbols of duplicated groups derived from Supplementary Fig. S4 were marked at the leading clades of each group. (**a**) The expression profiles were estimated using RNA-seq data from five organs at three developmental stages. Transcript levels are depicted using a colour scale indicating FPKM (fragments per kilobase of exon model per million mapped reads) values. The symbols representing development stages were as previously reported^31^. (**b**) The expression profiles were estimated using RNA-seq data from different grain cell types at three developmental stages. Transcript levels are depicted using a colour scale indicating the FPKM values. The symbols representing cell types and developmental stages were as previously reported^29^. (**c**) The expression profiles under heat (HS), drought (DS) and the combined stress of heat and drought (HS) after 1 and 6 h treatments^65^. Transcript levels are depicted using a colour scale indicating log_10_ values. The grey lines in panels a, b and c indicate that the *sHSP* genes were not detected in the corresponding RNA-seq dataset.

The expression profiles, as proxies for function, provide a valuable resource to study the evolutionary trajectories of duplicated genes^34^. To test the evolutionary patterns of sHSPs estimated by the above sequence analysis in this study, an integration transcriptional analysis was performed on published RNA-seq datasets from 28 hexaploid wheat samples, covering five tissues, three developmental stages and two types of abiotic stress (Fig. 6). In total, 95.7% (112 out of 117) of the identified sHSPs from bread wheat had an FPKM value higher than zero for at least one condition (6–15 samples for each condition), and were regarded as transcribed sHSPs, a much higher number of transcribed genes than in the wheat genome (71.4% on average)^41^. This result indicates that sHSPs (including expanded sHSPs) of hexaploid wheat are highly active and massive pseudogenization did not occurred after polyploidization (Fig. 6), potentially contributing to recent wheat genome evolution and, therefore, to the adaptive evolution of bread wheat.

The expression of these active sHSPs showed obvious spatiotemporal preferences (Fig. 6a,b). In general, the sHSPs were highly expressed at late developmental stages and in the corresponding tissues (spikes, filling grains and leaves after anthesis), which was consistent with the fact that frequent heat stress usually occurred after anthesis in most wheat growth areas^49^. The higher expression levels of sHSPs may enable rapid responses to heat stress or pre-heat stress. By contrast, some subfamilies displayed a constitutive expression pattern in all tissues and at all developmental stages. These subfamilies, which included MI, CIX and CV, showed low-level selection pressure and were less sensitive to heat induction. These results suggest that lower selection pressure may be related to the functional constrain shifts of constitutively expressed sHSPs.

As mentioned above, the duplicated groups of the CI, CV, ER, MI and MII subfamilies had undergone functional divergence. Consistent with this, the diverged duplicated groups of CI, ER, MI and MII showed distinct expression patterns (Fig. 6a,b, Supplementary Table S3). Only one group from each subfamily (ERa, MIa and MIIa) was found to be abundantly expressed in grain at 20 and 30 days post-anthesis. Similarly, in the CI subfamily, each of the duplicated groups could be further divided into two subgroups (CIaa, CIab and CIba, CIbb), and each subgroup showed distinct expression patterns. These results provide expression-level evidence of functional divergence between these duplicated groups. It should be noted that the expansion of hexaploid sHSPs occurs independently to the transcription patterns of these genes, i.e. less highly transcribed sHSPs are duplicated as frequently as more highly transcribed sHSPs.

Heat induction is a characteristic feature of sHSPs, and the RNA-seq data showed that 81.2% of sHSPs were markedly induced by heat (fold change ≥ 2 and false discovery rate (FDR) adjusted *p* < 0.01), a similar proportion as seen with *A. thaliana* and rice sHSPs (87–90%)^20, 22^. This finding indicates that expanded sHSPs in the current wheat genome maintain their conserved function in heat protection (Fig. 6c). Consistent with the different selection pressures and functional divergence, the expression patterns of different subfamilies and duplicated groups varied considerably under heat stress. The expression of CI, CII, P, ER, MII and Px, which were subject to stronger purifying selection, were up-regulated sharply, whereas the expression of CV, CIX and MI, which were subject to lower levels of purifying selection, were either not induced or only weakly induced by heat stress (Fig. 6c). The expression levels of duplicated groups of CI and MII also varied considerably. Among the expansion subfamilies of CI and CII, only CII (mainly possessing expansion on chromosome 3B) and parts of CI were strongly induced by heat stress (Fig. 6c), and similar transcriptional profiles were observed in grains. In fact, heat-induced sHSPs are also highly expressed in grains (Fig. 6), indicating that these sHSPs operate as a core heat response system in hexaploid wheat. In response to common abiotic stress, the majority of sHSPs showed strong heat induction, while a few were induced by drought stress, although similar effects resulted from both. To confirm the heat induction characters in transcriptional analysis, further qRT-PCR analysis showed that sHSPs in seedling leaves were rapidly induced by heat stress, and showed similar time-course heat response profiles (Supplementary Fig. S6 and Table S4), highlighting that sHSPs of bread wheat played their key roles in wheat adaptation to heat environments.

The transcriptional profiles and tissue preference of sHSPs displayed strikingly consistency with the evolutionary analysis based on sequences variations, further supporting the purifying selection and differential evolution. In addition, the duplicated sHSPs of CI and CII subfamilies kept similar transcripton patterns with original sHSPs (*t*-test *p* > 0.05, *p* = 0.1354 for CI and *p* = 0.1348 for CII, respectively), consistenting with the unaltered purifying seletions acted on them.

## Discussion

Here, we performed systematic sHSP evolution and expression analyses in Triticeae and revealed remarkable sHSP expansion along with wheat polyploidization. We also characterised the current sHSP status in bread wheat and hypothesised about future evolutionary patterns.

A previous study suggested that the genome size of hexaploid wheat was approximately the sum of its three diploid progenitors with 2–10% DNA loss^28^, indicating that, in general, each gene may be amplified three-fold in hexaploid wheat compared with their diploid progenitor. However, a recent study showed that the members of six gene families was considerably less than the sum of the three diploid progenitors^31^, suggesting that significant gene loss had occurred in these gene families with polyploidization, and some yet to be investigated gene families must have expanded to nearly triple the genome size of the diploid progenitors. In the current study, sHSPs were found to be dramatically expanded in hexaploid wheat, to an extent greater than merely the sum of its progenitors, which was consistent with the fact that stress response genes were over-represented with polyploidization in chromosome 3B^41^. These results suggested that the considerable expansion of a few gene families is also a common characteristic of wheat polyploidization, as well as the significant gene loss in specific gene families.

To date, only chromosome 3B has been sequenced completely^41^. The sHSP subfamily CII is dramatically expanded in this chromosome, and the sequence of its numbers share high similarity (Fig. 3a). Considering the short polyploidy history of tetraploid and hexaploid wheat^30^ and the corresponding high sequence similarity of expanded sHSPs, some expanded sHSPs with polyploidization may be undetectable by whole-genome or chromosome-based shotgun sequencing approaches, as previously reported^31^. Thus, the amounts of sHSPs in tetraploid and hexaploid wheat are likely to have been underestimated. Similarly, the degree of purifying selection acting on sHSPs may have been overestimated because of the limited numbers of mutations accumulated during the short evolutionary history of hexapolid wheat.

In this study, sHSPs duplications are specific occurred in subgenome A and B, but not D. The subgenome D, as a new member of hexaploid genome^30^, displayed quiet and stable status during the polyploidization. In addition, the sHSPs in subgenome D keep the conserved sequence and classification with their diploid progenitor, and show similar purifying selection pressure with its diploid progenitors. With the improvement of wheat genomics, serveral conclusions about subgenomic biases in wheat development and stress reponses were reported, such as the cell type and stage dependent subgenome dominance in wheat grain development^29^, the bias towards D subgenome in response to *Fusarium* head blight^50^, towards B and D subgenomes in response to *Fusarium pseudograminearum* ^51^.

Diploidization makes the loss of duplicated genes and returns the most fractions of a polyploid genome back to the singleton state^32^. Our result shows that the genome size of current tetraploid is close to the total of its two diploid progenitors, along with 13.1% (AA^uu^ and S^sh^S^sh^ to AABB^Tdi^)–31.2% (AA^uu^ and SS to AABB^Tdc^) gene loss (Fig. 1b), suggesting that the tetraploid genome has not experienced or is still undergoing the diploidization process. However, the number of sHSPs in tetraploid wheat was similar to that in its diploid progenitors, rather than reflecting the sum of the two diploid progenitors (Fig. 1b), indicating that a remarkable number of sHSPs had been lost in tetraploid wheat, likely has experienced diploidization process. In addition, it is reported that the allotetraploidzation in wheat rapidly induced extremely biased homeolog expression and this trend is further enhanced in the subsequential domestication and evolution of polyploid wheats, providing an indirect evidence for the diploidization of tetraploid wheat^34, 52^, and supporting the diploidisation may be responsible for unusual less sHSPs in tetraploid wheat. However, more genomic complexity and incomplete genome sequences of tetraploid wheat still be the potential reasons for the smaller number of sHSPs in tetraploid.

Although both inter- and intrachromosomal duplication rates are apparently higher in wheat than in other grass species^31, 41^, expanded sHSPs in hexaploid wheat mainly resulted from intrachromosomal duplications in this study, highlighting the role of segmental or tandem duplications in gene expansion with wheat polyploidization. Interestingly, genes involved in the stress response were also identified in the typically overrepresented gene category in chromosome 3B^41^, indicating that sHSP expansion may not occurred at random, but instead occurred specifically with wheat polyploidization. It will be intriguing to investigate why stress response genes particularly expanded, as well as the mechanism by which organisms expand specific genes by intrachromosomal duplications during polyploidization.

Selection pressure is the key factor driving speciation and genetic evolution. In this study, we revealed that purifying selection and positive selection acted via a conserved pattern on sHSPs across members of Triticeae, indicating that the sHSPs of hexaploid wheat, despite high redundancy, adhere to the common evolutionary rules of Triticeae sHSPs. The weaker levels of purifying selection and positive selection on the N-terminal region of sHSPs may allow for greater functional plasticity, potentially allowing these proteins to adjust to their substrates, preventing or reversing the unnatural substrate aggregations usually caused by heat stress^3, 4, 7–11^. For hexaploid wheat, this may be a critical step for sHSP evolution in polyploidization, because sHSPs may not only need to recognize the original substrates from the same subgenome, but also need to recognize the homolog or heterolog substrates from other subgenomes, which may be critical for future diploidization.

Owing to the dosage and functional redundancy of sHSPs in wheat, greater complexity and diversity at the evolutionary and functional level were observed than the sHSPs in related species (Figs 1, 2 and 6). Theoretically, this redundancy could provide a larger reservoir for genetic variation, facilitating the evolution of new sHSPs or novel functions, a more efficient response to heat stress and a stronger adaptability to wide range of climatic conditions, which was assumed to be a key factor in the success of wheat as a global food crop^31^. Paradoxically, bread wheat is actually heat vulnerable^25, 53–55^. This fact suggests that hexaploid wheat thermotolerance can’t be enhanced by simply increasing sHSPs amount. However, the expansion of sHSPs prefer provide massive genome resources to compensate the weak thermotolerance. This hypothesis may provide a logic that why stress response genes prefer be expanded with polyploidization^41^. On the other hand, due to the early polyploidization status of bread wheat^31^, the interaction between wheat genome and environmental changes and the acquirement of heat adaption may be ongoing.

Most of the expanded sHSPs in hexaploid wheat are likely to be lost in the diploidization process when it adapts to its habitat and corresponding environment^28^, as demonstrated by our findings in the current diploid and tetraploid relatives. Considering that global warming is an unprecedented event in the evolutionary history of Triticeae, the future evolution of sHSPs is difficult to predict. However, it is clear that hexaploid wheat has evolved a massively expanded number of active sHSPs with polyploidization, and is ready to action in new heat environments.

In summary, our study presents the evidence of the considerable expansion of sHSPs by intrachromosomal duplications in hexaploid wheat. Of these abundant sHSPs, the ACD domain is under strong purifying selection to maintain conserved function, while the highly variable N-terminal region confers greater plasticity for substrates binding to response to genomic and environmental changes. Most sHSPs maintain active transcription and the ability for heat induction. We speculat that the expansion of active sHSPs with wheat polyploidization confers extra gene resource for the future adaption evolution of bread wheat, like the sHSPs dunplications in CI, CV, ER, MI and MII subfamilies, subsequently functional divergence, and corresponding expression variations occurred before wheat polyploidization. Wheat researchers and breeders may therefore make full use of sHSP resources to improve wheat heat tolerance in the face of global warming. Our findings also provide insight into the intrachromosomal duplications, gene expansions and environmental adaptation processes that occurred along with plant polyploidization.

## Methods

### Data collection and identification of Triticeae sHSPs

The bread wheat genome sequences and annotation information were downloaded from the EnsemblPlants database (ftp://ftp.ensemblgenomes.org/pub/plants/release-26/fasta/triticum_aestivum/dna/). The genomes and gene predictions of the wheat diploid and tetraploid relatives, *Brachypodium distachyon* and *Hordeum vulgare*, were obtained from PGSB PlantsDB (ftp://ftpmips.helmholtz-muenchen.de/plants/)^31, 56^. First, the keywords “alpha crystallin protein” and “small heat shock protein” were searched against each genome annotation to predict candidate *sHSP* genes. Then, we used the Hmmsearch program in HMMER^57^ to search for the family-specific HMM profiles of sHSPs (PF00011) downloaded from the Pfam database (http://pfam.xfam.org/)^58^, using each of the predicted proteomes as queries. These two results were merged to remove redundancy and examined for the ACD in the InterPro (http://www.ebi.ac.uk/interpro/) and PROSITE (http://prosite.expasy.org/) databases. The sequences in which ACD domain were detected in InterPro or PROSITE, were aligned using the M-Coffee program, which combines the output of popular alignment programs^59^. A phylogenetic tree was constructed based on the ACD using the neighbour-joining method and a bootstrap test of 1000 iterations, and the tree was presented by iTOL^60^. Triticeae sHSPs were grouped into different subfamilies by phylogenetic analysis using sHSPs from *A. thaliana* and rice as markers. Wheat sHSPs were mapped to the chromosome based on the annotation information and presented by Circos^61^.

### Molecular evolution analysis

The codeml program from the PAML4.7 package^46^ was used to estimate the ω values for Triticeae relatives and sHSP subfamilies. One-ratio model (model 0) which assumes a constant ω ratio along all branches and two-ratio model (model 2), which allows different ω ratios between foreground and background lineages, were used to detect different selective pressures between the duplicated clades. The site-specific discrete model (model 3), which assumes two classes of sites with different ω ratios, and the clade model (model D), which allows selective pressure at one class of sites (foreground clade) to be different from the rest of the phylogeny, were used to detect functional divergence after gene duplication. The branch-site model A (model A), which assumes one class of sites in the foreground lineage ω > 1 and null model A, with fixed ω = 1, were compared to detect positive selection in specific lineages. In PAML analysis, the protein sequence alignments were converted into corresponding codon alignments using the PAL2NAL program^62^. The Selecton program^63^ was used to detect the selective pressure acting on each amino acid residue and the SPEER-SERVER^64^ was used to identify the type I and type II residues that contribute to functional divergence between duplicated clades.

### Expression profile analysis of wheat sHSPs

We performed expression profiling analysis of wheat sHSPs using high-throughput RNA-seq data. The RNA-seq data from five tissues (leaf, root, grain, spike and stem) at three development stages were obtained from URGI (https://urgi.versailles.inra.fr/files/RNASeqWheat/)^31^, and the RNA-seq data from different cell types of grain at three development stages were downloaded from NCBI (Accession: PRJEB5135)^29^. RNA-seq data from wheat seedlings under heat and drought treatments were downloaded from NCBI (PRJNA257316)^65^. The quality of the public RNA-seq data was checked with the FastQC program (http://www.bioinformatics.babraham.ac.uk/projects/fastqc/). The high-quality paired-end RNA-seq reads from each library were aligned to the wheat reference genome (*Triticum aestivum* IWGSC v2.26; ftp://ftp.ensemblgenomes.org/pub/plants/release-26/fasta/triticum_aestivum/dna/) using hisats v2.0.0^66^ at “very-sensitive” preset parameters in the “end-to-end” mode. The intron length was set to 20 to 5000 nucleotides during alignment. Alignments between reads and wheat reference genome sequences were input into stringTie v1.1.1^67^ for the normalization and estimation of the gene expression level. Gene expression levels were reported as fragments per kilobase of exon model per million mapped reads (FPKM). A gene was regarded as expressed in a sample if the FPKM was greater than zero. The wheat genome annotation information used in these analyses was obtained from the EnsemblPlants database (ftp://ftp.ensemblgenomes.org/pub/plants/release-26/gff3/triticum_aestivum). The expression data were presented by HemI^68^ and iTOL^60^.

### Quantitative reverse transcriptase polymerase chain reaction (qRT-PCR) validation

Total RNA was isolated from the leaves of 7-day seedlings of the hexaploid wheat variety *Chinese Spring*, which had been subjected to heat stress, using a RNA extraction kit with TRIzol reagent (Tiangen Biotech, Beijing, China). A FastQuant RT kit (Tiangen Biotech, Beijing, China) was used to synthesize cDNA from the treated RNA with an oligo-dT primer in a 50-μl reaction. Real-time quantitative PCR primers were designed in Primer Premier 5.0 software. The CDS sequence of bread wheat is downloaded from the Ensemble database (ftp://ftp.ensemblgenomes.org/pub/release-26/plants/fasta/triticum_aestivum/cds/). The size of the PCR product was set to 100–200 bp and the length of the primer was 15–25 bp. A pre-PCR was performed using wheat cDNA as a template, and primer produced a single specific band was further used for real-time PCR. For PCR, 0.1–0.25 μM cDNA were amplified with specific primers for each of eight different gene models, with the 1 × SYBR Green Master Mix Kit (Applied Biosystems, Foster City, CA, USA) in a final volume of 12.5 μL.

## Electronic supplementary material

Supplementary Figures and Tables
