# Supplementary material for: Massive expansion and differential evolution of small heat shock proteins with wheat (Triticum aestivum L.) polyploidization
Source: Sci Rep. 2017 May 31;7:2581. doi: 10.1038/s41598-017-01857-3 (PMC5451465; doi:10.1038/s41598-017-01857-3)
Supplement: Supplementary file 1 — Supplementary Figures and Tables [file 41598_2017_1857_MOESM1_ESM.pdf]

Massive expansion and differential evolution of small heat shock proteins with wheat (*Triticum aestivum* L.) polyploidization

Xiaoming Wang[1], Ruochen Wang[1], Chuang Ma[2], Zhenshan Liu[1], Zhonghua Wang[1], Xue Shi[1], Jun Cao[3], Shengbao Xu[1*], Qixin Sun[1,4*]

Xiaoming Wang[1], wangxm@nwsuaf.edu.cn; Ruochen Wang[1], wangruochen@nwsuaf.edu.cn; Chuang Ma[2], chuangma2006@gmail.com; Zhenshan Liu[1], zhenshanLiu@nwsuaf.edu.cn; Zhonghua Wang[1], zhonghuawang@nwsuaf.edu.cn; Xue Shi[1], shixue@nwsuaf.edu.cn; Jun Cao[3], archangel@nwsuaf.edu.cn; Shengbao Xu[1], xushb@nwsuaf.edu.cn; Qixin Sun[1,4], sunqx@nwsuaf.edu.cn

Author affiliations:

1. State Key Laboratory of Crop Stress Biology for Arid Areas, College of Agronomy, Northwest A&F University, Yangling, Shaanxi, 712100, China.
2. College of Life Sciences, Northwest A&F University, Shaanxi, 712100, China.
3. Innovation Experimental College, Northwest A&F University, Shaanxi, 712100, China.
4. Department of Plant Genetics & Breeding, China Agricultural University, Yuanmingyuan Xi Road No. 2, Haidian District, Beijing, 100193, China.

* **Correspondence:** Shengbao Xu, xushb@nwsuaf.edu.cn Tel.: 8618706867411

Qixin Sun, sunqx@nwsuaf.edu.cn Tel.: 8602987086023

Supplementary legends

Figure. S1. Phylogenetic analysis of the *sHSP* gene family in Triticeae. The tree was constructed using the neighbour-joining method with bootstrap analysis (1000 iterations) based on the alignment of the amino acid sequences of the conserved α-crystallin domain. The defined classification of sHSPs in rice and *A. thaliana* are used as markers and the subfamily symbols are depicted on the outer layer. The bootstrap values are plotted as circles at the nodes, with the circle size proportional to the bootstrap value. Abbreviations: At, *A. thaliana*; Os, *Oryza sativa*; Bradi, *Brachypodium distachyon*; M and AK, *Hordeum vulgare*; Aet, *Aegilops tauschii*; Asp, *Aegilops speltoides*; Ash, *Aegilops sharonensis*; Tm, *Triticum monococcum*; Tu, *Triticum urartu*; Tdi, *Triticum durum*, cv. Cappelli; Tdc, *Triticum durum*, cv. Strongfield; Traes, *Triticum aestivum*.

Figure. S2. Circos representation of wheat sHSP chromosome locations. Grey bars represent each chromosome, labelled 1A, 1B, etc. Outer numbers show the scale per million bases (Mb). Only chromosome fragments that contain *sHSP* genes are shown, and fragments from the same chromosome are bridged with red bars. Grey lines following each chromosome represent unanchored sequence scaffolds, which contain *sHSP* genes. Coloured bars within chromosomes represent *sHSP* genes. Members of the same subfamily are depicted by the same colour and are connected in the centre by the corresponding colour. The relationships between subfamilies and colours are: CI, dark green; CII, dark red; CIII, dark yellow; CV, brown; CVIII, lime; CIX, orange; CX,

black; ER, purple; MI, dark blue; MII, light blue; P, dark pink; Px, yellow; Acd, grey.

Figure. S3. Copy number variation of sHSP subfamily during wheat polyploidization. Abbreviations, At, *Arabidopsis thaliana*; Os, *Oryza sativa*; Bd, *Brachypodium distachyon*; Hv, *Hordeum vulgare*; AA^{mm}, *Triticum monococcum*; AA^{uu}, *Triticum urartu*; $S^{sh}S^{sh}$, *Aegilops sharonensis*; SS, *Aegilops speltoides*; DD, *Aegilops tauschii*; $AABB^{Tdc}$, *Triticum durum*, cv. Strongfield; $AABB^{Tdi}$, *Triticum durum*, cv. Cappelli; Ta^{AA}, subgenome A of bread wheat; Ta^{BB}, subgenome B of bread wheat; Ta^{DD}, subgenome D of bread wheat; Ta, *Triticum aestivum*.

Figure. S4. Unrooted trees used for PAML analysis, including asymmetric evolution, functional divergence and positive selection detection. The different groups in each subfamily are depicted on different coloured backgrounds. Abbreviations: At, *A. thaliana*; Os, *Oryza sativa*; Bradi, *Brachypodium distachyon*; M and AK, *Hordeum vulgare*; Aet, *Aegilops tauschii*; Asp, *Aegilops speltoides*; Ash, *Aegilops sharonensis*; Tm, *Triticum monococcum*; Tu, *Triticum urartu*; Tdi, *Triticum durum*, cv. Cappelli; Tdc, *Triticum durum*, cv. Strongfield; Traes, *Triticum aestivum*.

Figure. S5. Protein alignments of the CI, CV, CIX, ER, MI and MII subfamilies. For the group names of each subfamily, see Supplementary Fig. S4. Abbreviations: At, *A. thaliana*; Os, *Oryza sativa*; Bradi, *Brachypodium distachyon*; M and AK, *Hordeum vulgare*; Aet, *Aegilops tauschii*; Asp, *Aegilops speltoides*; Ash, *Aegilops sharonensis*; Tm, *Triticum monococcum*; Tu, *Triticum urartu*; Tdi, *Triticum durum*, cv. Cappelli; Tdc, *Triticum durum*, cv. Strongfield; Traes, *Triticum aestivum*.

Figure. S6. Real time PCR analysis of sHSP expression at different heat treatment times. Total RNA from the leaves of 7-day-old seedlings of the bread wheat variety *Chinese Spring* were used for PCR analysis.

Table S1. Number of each sHSP subfamily in different species.

Table S2. Estimates from the codon-substitution evolution models for duplicated groups in the CI, CV, CIX, ER, MI and MII subfamilies.

Table S3. Statistical test of expression patterns between duplicated groups in CI, ER, MI and MII subfamilies.

Table S4. The primers unsed for Real time PCR.

Supplementary Figure S1

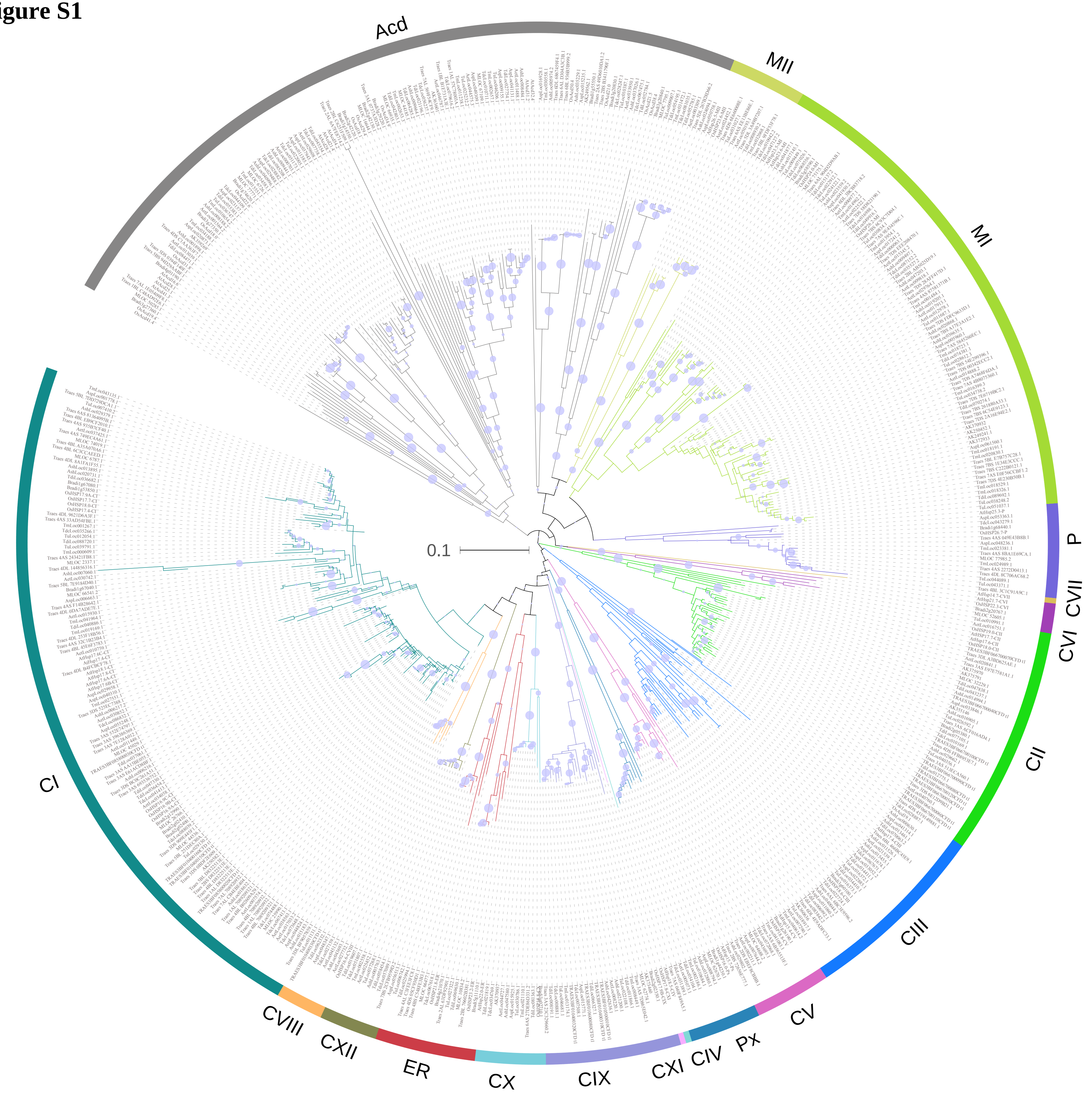

Supplementary Figure S2

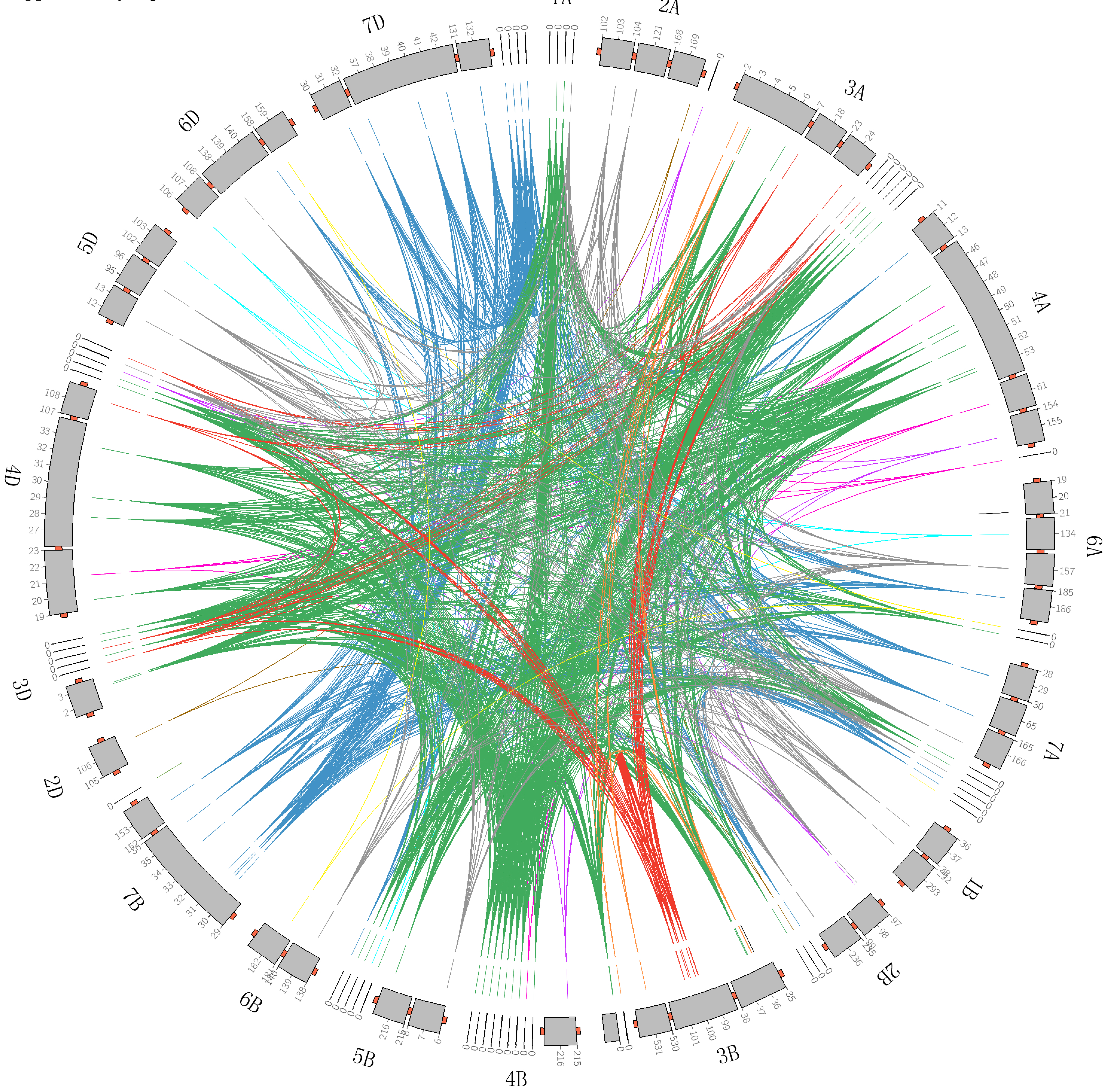

Supplementary Figure S3

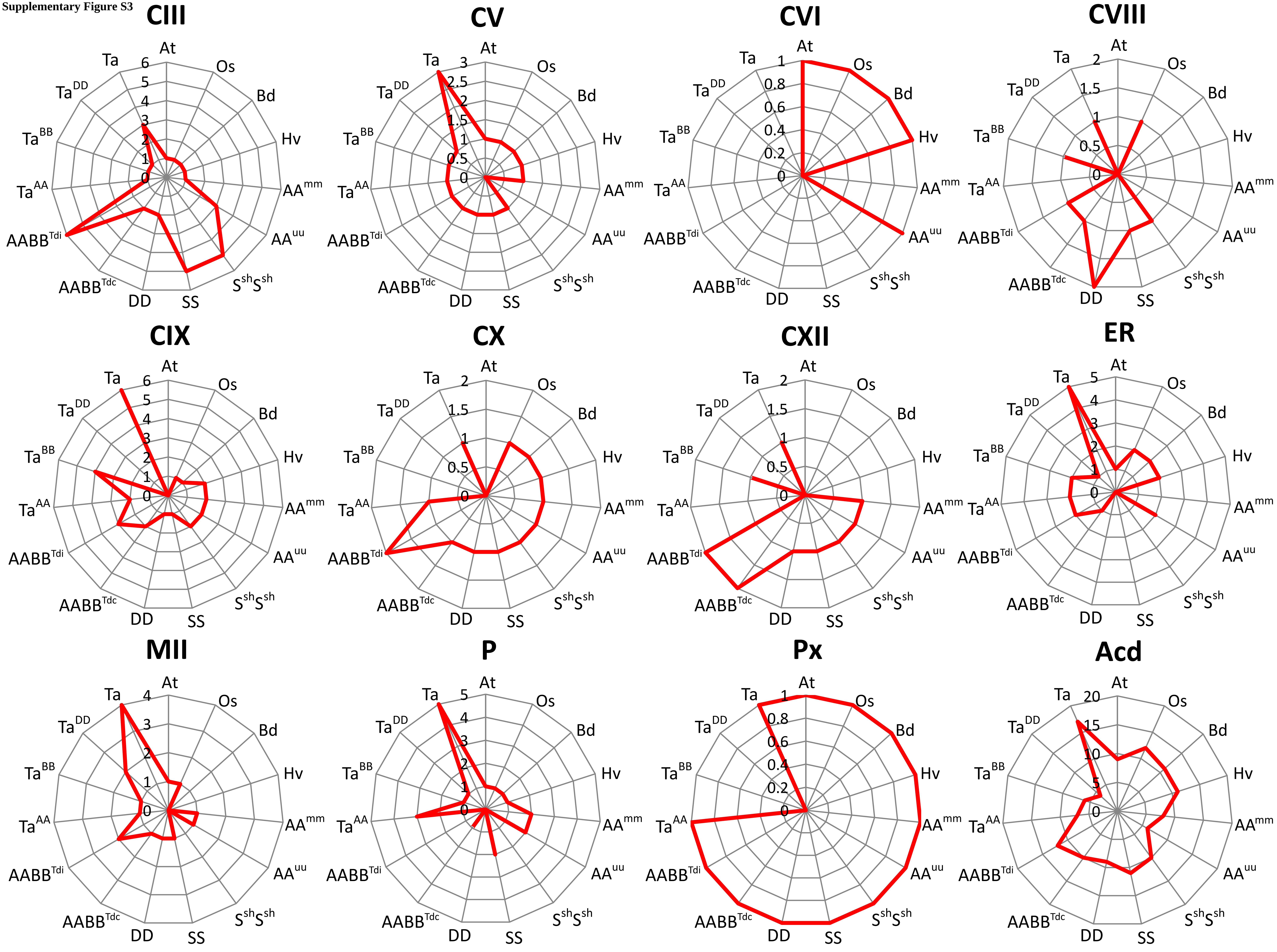

Supplementary Figure S4

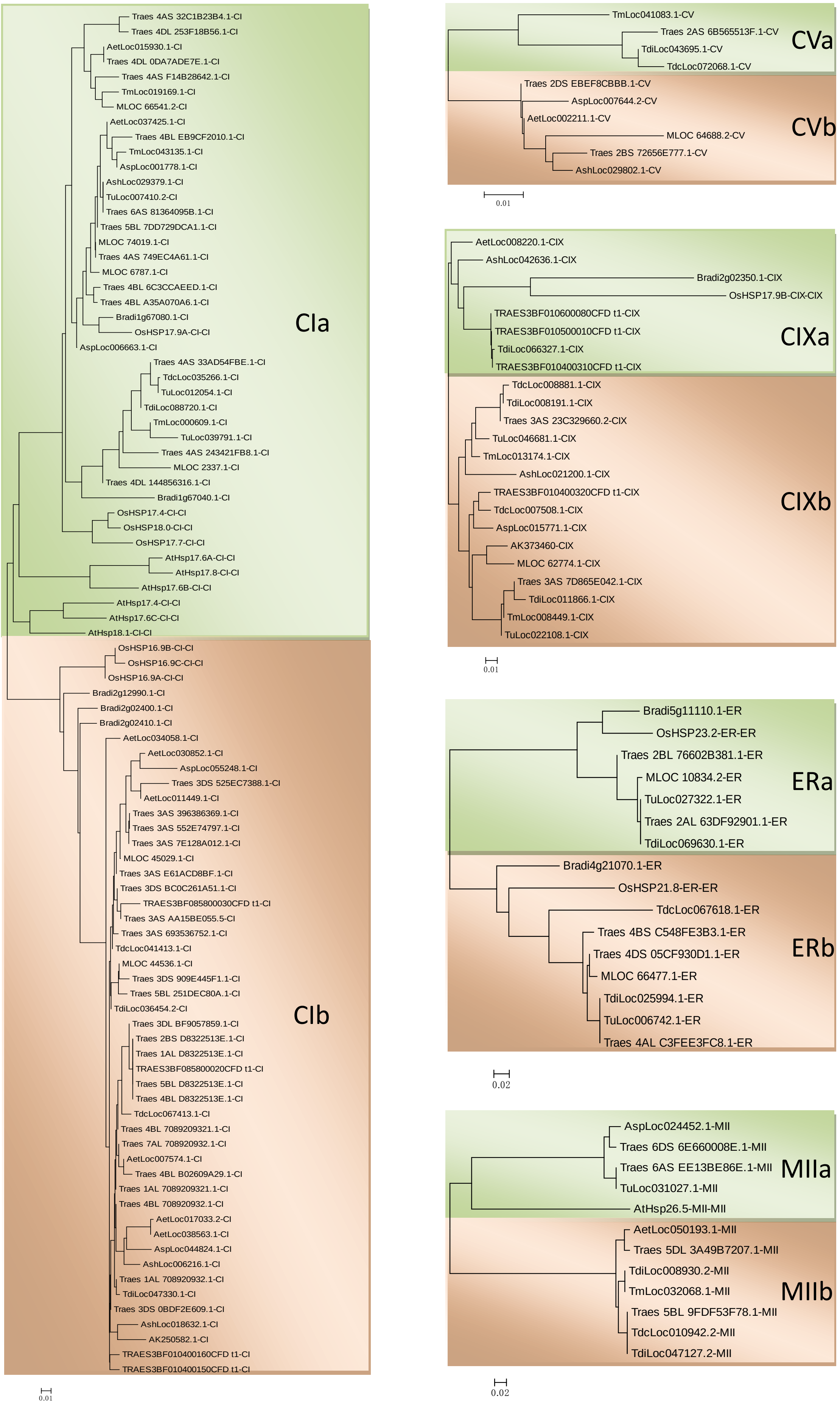

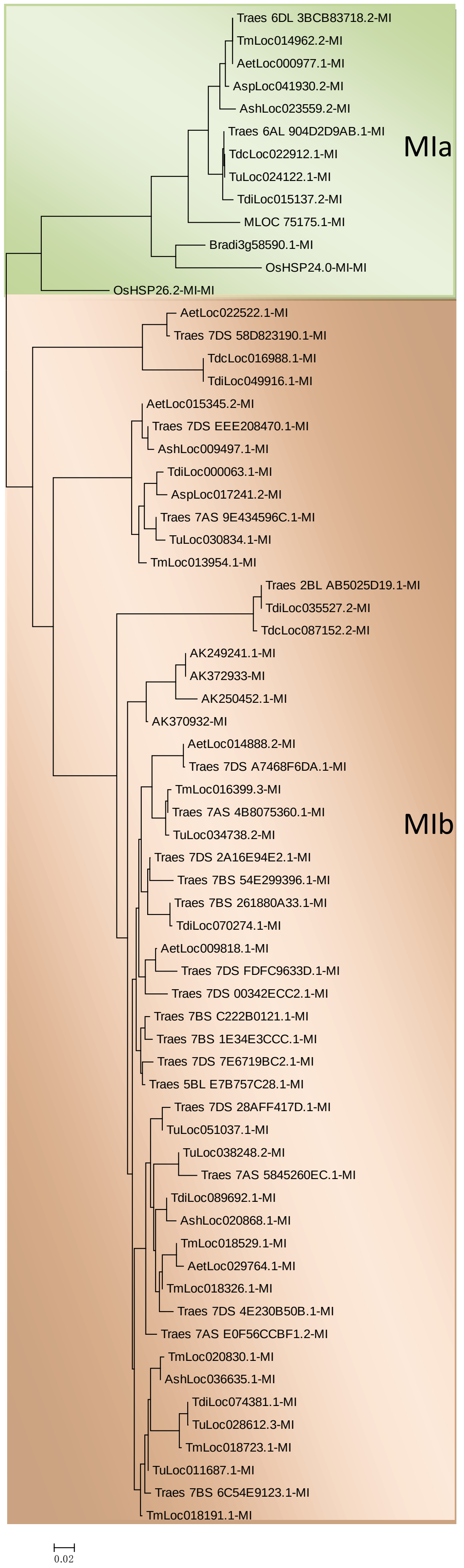

Traes 6DL 3BCB83718.2-MI
TmLoc014962.2-MI
AetLoc000977.1-MI
AspLoc041930.2-MI
AshLoc023559.2-MI
Traes 6AL 904D2D9AB.1-MI
TdcLoc022912.1-MI
TuLoc024122.1-MI
TdiLoc015137.2-MI
MLOC 75175.1-MI
Bradi3g58590.1-MI
OsHSP24.0-MI-MI
OsHSP26.2-MI-MI
MIa
AetLoc022522.1-MI
Traes 7DS 58D823190.1-MI
TdcLoc016988.1-MI
TdiLoc049916.1-MI
AetLoc015345.2-MI
Traes 7DS EEE208470.1-MI
AshLoc009497.1-MI
TdiLoc000063.1-MI
AspLoc017241.2-MI
Traes 7AS 9E434596C.1-MI
TuLoc030834.1-MI
TmLoc013954.1-MI
Traes 2BL AB5025D19.1-MI
TdiLoc035527.2-MI
TdcLoc087152.2-MI
AK249241.1-MI
AK372933-MI
AK250452.1-MI
AK370932-MI
AetLoc014888.2-MI
Traes 7DS A7468F6DA.1-MI
TmLoc016399.3-MI
Traes 7AS 4B8075360.1-MI
TuLoc034738.2-MI
MIb
Traes 7DS 2A16E94E2.1-MI
Traes 7BS 54E299396.1-MI
Traes 7BS 261880A33.1-MI
TdiLoc070274.1-MI
AetLoc009818.1-MI
Traes 7DS FDFC9633D.1-MI
Traes 7DS 00342ECC2.1-MI
Traes 7BS C222B0121.1-MI
Traes 7BS 1E34E3CCC.1-MI
Traes 7DS 7E6719BC2.1-MI
Traes 5BL E7B757C28.1-MI
Traes 7DS 28AFF417D.1-MI
TuLoc051037.1-MI
TuLoc038248.2-MI
Traes 7AS 5845260EC.1-MI
TdiLoc089692.1-MI
AshLoc020868.1-MI
TmLoc018529.1-MI
AetLoc029764.1-MI
TmLoc018326.1-MI
Traes 7DS 4E230B50B.1-MI
Traes 7AS E0F56CCBF1.2-MI
TmLoc020830.1-MI
AshLoc036635.1-MI
TdiLoc074381.1-MI
TuLoc028612.3-MI
TmLoc018723.1-MI
TuLoc011687.1-MI
Traes 7BS 6C54E9123.1-MI
TmLoc018191.1-MI
0.02

Supplementary Figure S5

```
                                                                                                      1         10          20
Traes_4AS_32C1B23B4.1-CI     .......................................................................MSLIPRGK..AFDPFSL.DLTDPF
Traes_4DL_253F18B56.1-CI     .......................................................................MSLIPRGK..AFDPFSL.DLSDPF
AetLoc015930.1-CI            ....................................................................KDAPDPSPSRP..RQSETTK.NLWDPF
Traes_4DL_0DA7ADE7E.1-CI     .......................................................................MSLIPRGN..AFDPFSL.DLWDPF
Traes_4AS_F14B28642.1-CI     .......................................................................MSLIPRGN..AFDPFSP.DLWDPF
TmLoc019169.1-CI             ...............................................................................................
MLOC_66541.2-CI              .......................................................................MSLIPRGN..AFDPFSV.DLWNPF
AetLoc037425.1-CI            ...............................................................................................
Traes_4BL_EB9CF2010.1-CI     ...............................................................................................
TmLoc043135.1-CI             ...............................................................................................
AspLoc001778.1-CI            ...............................................................................................
AshLoc029379.1-CI            ...........................TQESL..KKAPIKELQQINRSPFRFQYQPNCRFIPHQQLESPPTMSLIRCSN..VFDPFSL.DFFDPF
TuLoc007410.2-CI             .........................RIYSQKE..HRSKSSQKSIDHPSASNTSQIVDSFHQHLESPPIRPTMSLIRRSN..VFDPFSL.DFFDPF
Traes_6AS_81364095B.1-CI     ...............................................................................................
Traes_5BL_7DD729DCA1.1-CI    ...............................................................................................
MLOC_74019.1-CI              .......................................................................MSLIRRSN..VFDPFSL.DFFDPF
Traes_4AS_749EC4A61.1-CI     .......................................................................MSLIRRSN..VFDPFSL.DFFDPF
MLOC_6787.1-CI               .......................................................................MSLIRRSN..VFDPFSL.DFFDPF
Traes_4BL_6C3CCAEED.1-CI     .......................................................................MSLIRRSN..VFDPFSL.DLWDPF
Traes_4BL_A35A070A6.1-CI     .......................................................................MSLIRRSN..VFDPFSL.DFFDPF
Bradi1g67080.1-CI            .......................................................................MSLIRRSN..VFDPFSL.DLWDPF
OsHSP17.9A-CI-CI             .......................................................................MSLIRRSN..VFDPFSL.DLWDPF
AspLoc006663.1-CI            ...............................................................................................
Traes_4AS_33AD54FBE.1-CI     .......................................................................MSLIRRGD..VFDPFSL.DLWDPF
TdcLoc035266.1-CI            ...............................................................................................
TuLoc012054.1-CI             ...............................................................................................
TdiLoc088720.1-CI            ...............................................................................................
TmLoc000609.1-CI             .......................................................................MSLIRRGD..VFDPFSL.DLWDPF
TuLoc039791.1-CI             ...............................................................................................
Traes_4AS_243421FB8.1-CI     .......................................................................MSLIRRGD..VFDPFSL.DLWDPF
MLOC_2337.1-CI               .......................................................................MSLIRRGD..VFDPFSL.DLWDPF
Traes_4DL_144856316.1-CI     .......................................................................MSLIRRGD..VFDPFSL.DLWDPF
Bradi1g67040.1-CI            .......................................................................MSLIRRGD..VFDPFSL.DLWDPF
OsHSP17.4-CI-CI              .......................................................................MSMIRRSN..VFDPFSL.DLWDPF
OsHSP18.0-CI-CI              .......................................................................MSLIRRSN..VFDPFSL.DLWDPF
OsHSP17.7-CI-CI              .......................................................................MSLIRRGN..AFDPFSL.DLWDPV
AtHsp17.6A-CI-CI             ................................................................MSLIPSFFGNNRRINNNIFDPFSL.DVWDPF
AtHsp17.8-CI-CI              ................................................................MSLIPSFFGNNRRS.NSIFDPFSL.DVWDPF
AtHsp17.6B-CI-CI             ................................................................MSMIPSFFNNNRR..SNIFDPFSL.DVWDPF
AtHsp17.4-CI-CI              ................................................................MSLV.PSFFGGRRTN..VFDPFSL.DVWDPF
AtHsp17.6C-CI-CI             ................................................................MSLI.PSIFGGRRTN..VFDPFSL.DVFDPF
AtHsp18.1-CI-CI              ................................................................MSLI.PSIFGGRRSN..VFDPFSQ.DLWDPF
OsHSP16.9B-CI-CI             .......................................................................MSLVRRSN..VFDPFSLDLW.DPF
OsHSP16.9C-CI-CI             .......................................................................MSLVRRSN..VFDPFAD.FW.DPF
OsHSP16.9A-CI-CI             .......................................................................MSLVRRSN..VFDPFSLDLW.DPF
Bradi2g12990.1-CI            .......................................................................MSLVRRS...AFDPFAD.FW.DPL
Bradi2g02400.1-CI            .......................................................................MSLVRRSS..VFDPRSD.FWFDPM
Bradi2g02410.1-CI            .......................................................................MSLVRRGS..VFDPFSQDLW.DPI
AetLoc034058.1-CI            .........................................................................IVRRSN..VFDPFAD.LWADPL
AetLoc030852.1-CI            ...............................................................................................
AspLoc055248.1-CI            XXXXXXXXX..............XXXXXXXXX..XXXXXXXXXXXXXXXXXXXXXXXXXXXXXXXXXXXXXXXXXXXXX..XXXXXXXXXX.XXX
Traes_3DS_525EC7388.1-CI     ...............................................................................................
AetLoc011449.1-CI            ......................................................................AMSIVRRSN..VFDPFAD.LWADPF
Traes_3AS_396386369.1-CI     ...............................................................................................
Traes_3AS_552E74797.1-CI     ...............................................................................................
Traes_3AS_7E128A012.1-CI     .......................................................................MSIVRRSN..VFDPFAD.LWADPF
MLOC_45029.1-CI              ...MSSSLT..............SHRPPSQPK.........PVPSAKASSIQRSDTEFSERTSLSNQSTPTMSIVRRSN..VFDPLAG.LWADPF
Traes_3AS_E61ACD8BF.1-CI     M.ETRRSSWSQTLIKCRVVEMLQEQHLRRYIS..GLPSPCTPTQISFKQKQTTSNDPQNFPSAQAEAINTTMSIVRRSN..VFDPFAD.LWADPF
Traes_3DS_BC0C261A51.1-CI    .......................................................................MSIVRRSN..VFDPFAD.LWADPF
TRAES3BF085800030CFD_t1-CI   .......................................................................MSIVRRSN..VFDPFAD.LWADPF
Traes_3AS_AA15BE055.5-CI     AQNRNKEKL..............VEPR.......................................WKPKDDQTLIDCR..VLETLQEHWK.K..
Traes_3AS_693536752.1-CI     ...............................................................................................
TdcLoc041413.1-CI            .........................................................................MVRRSN..VFDPFAD.LWADPF
MLOC_44536.1-CI              .......................................................................MSIVRRSN..AFDPFAD.LWADPF
Traes_3DS_909E445F1.1-CI     ....................................................................................PFAD.LWADPF
Traes_5BL_251DEC80A.1-CI     RSSTRRSFTGETSRRFKSTTRRRYKYVASHRPPFQLKPVSSKSKQTASCSYLRSDTEFFFRAHKPKQSAPTMSMVRRSN..VFDPFAD.LWADPF
TdiLoc036454.2-CI            .........................................................................MVRRSN..VFDPFAD.LWADPF
Traes_3DL_BF9057859.1-CI     ...............................................................................................
Traes_2BS_D8322513E.1-CI     .......................................................................MSIVRRSN..VFDPFAD.LWADPF
Traes_1AL_D8322513E.1-CI     .......................................................................MSIVRRSN..VFDPFAD.LWADPF
TRAES3BF085800020CFD_t1-CI   .......................................................................MSIVRRSN..VFDPFAD.LWADPF
Traes_5BL_D8322513E.1-CI     .......................................................................MSIVRRSN..VFDPFAD.LWADPF
Traes_4BL_D8322513E.1-CI     .......................................................................MSIVRRSN..VFDPFAD.LWADPF
TdcLoc067413.1-CI            ...............................................................................................
Traes_4BL_7089209321.1-CI    ...............................................................................................
Traes_7AL_708920932.1-CI     ...............................................................................................
AetLoc007574.1-CI            ...............................................................................................
Traes_4BL_B02609A29.1-CI     .......................................................................D.................KWHR..
Traes_1AL_7089209321.1-CI    ...............................................................................................
Traes_4BL_708920932.1-CI     ...............................................................................................
AetLoc017033.2-CI            ...............................................................................................
AetLoc038563.1-CI            ...............................................................................................
AspLoc044824.1-CI            ...............................................................................................
AshLoc006216.1-CI            ...........VDTNQTRSKYQASRRPPSQVP..NSRQCQAKANNFPFLPTIQVQYTESSPSYKPKQATPAMSIVRRSN..VFDPIAD.LWVDPF
Traes_1AL_708920932.1-CI     ...............................................................................................
TdiLoc047330.1-CI            .........................................................................IVRRSN..VFDPFAD.LWADPF
Traes_3DS_0BDF2E609.1-CI     ............................................................................RSN..VFDPFAD.LWADPF
AshLoc018632.1-CI            ...............................................................................................
AK250582.1-CI                .......................................................................MSIVRRSN..VLDPFAD.LWADPF
TRAES3BF010400160CFD_t1-CI   .......................................................................MSIVRRSN..VFDPFAD.LWADPF
TRAES3BF010400150CFD_t1-CI   .......................................................................MSIVRRSN..VFDPFAD.LWADPF
```

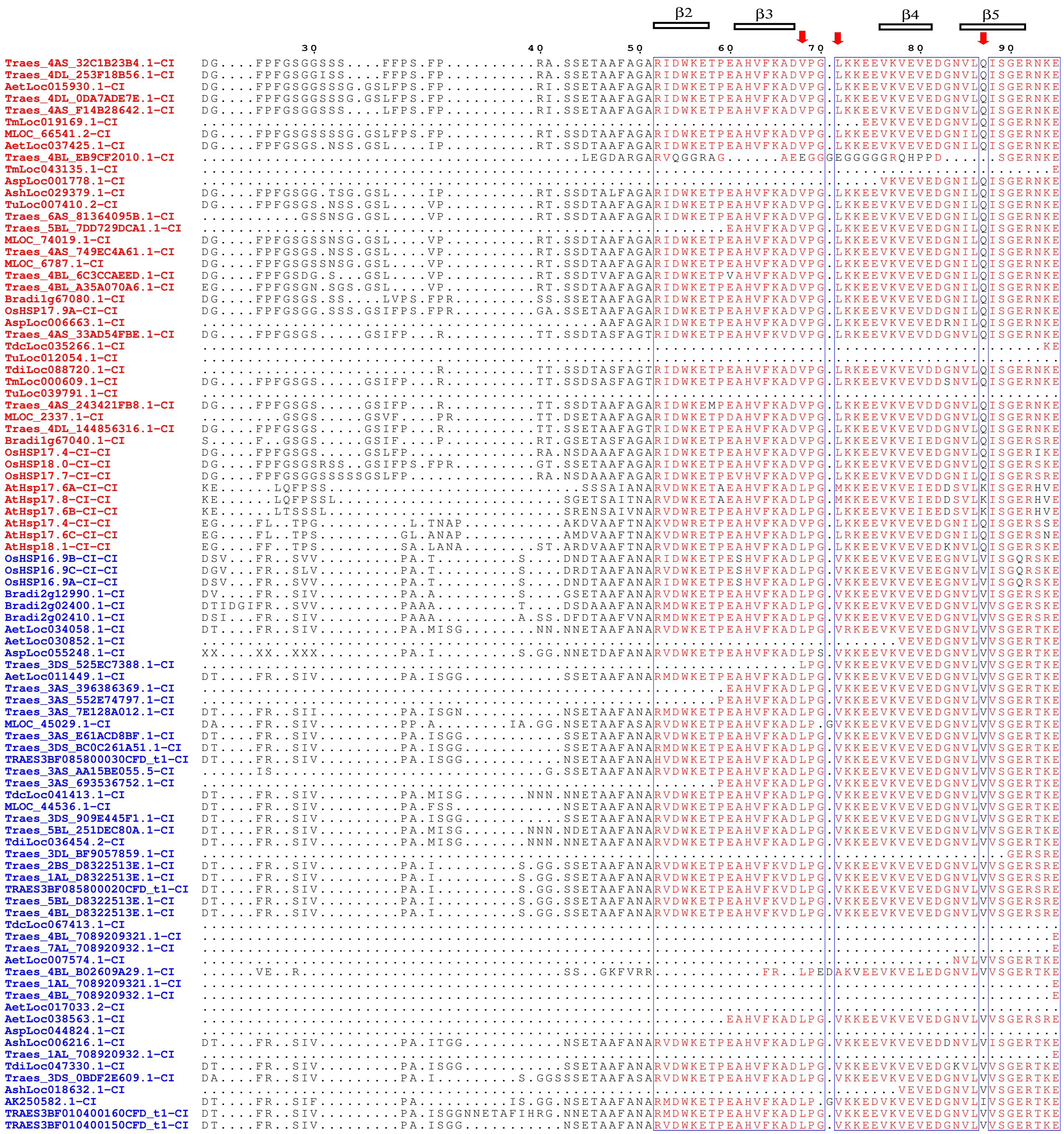

β2
β3
β4
β5
30
40
50
60
70
80
90

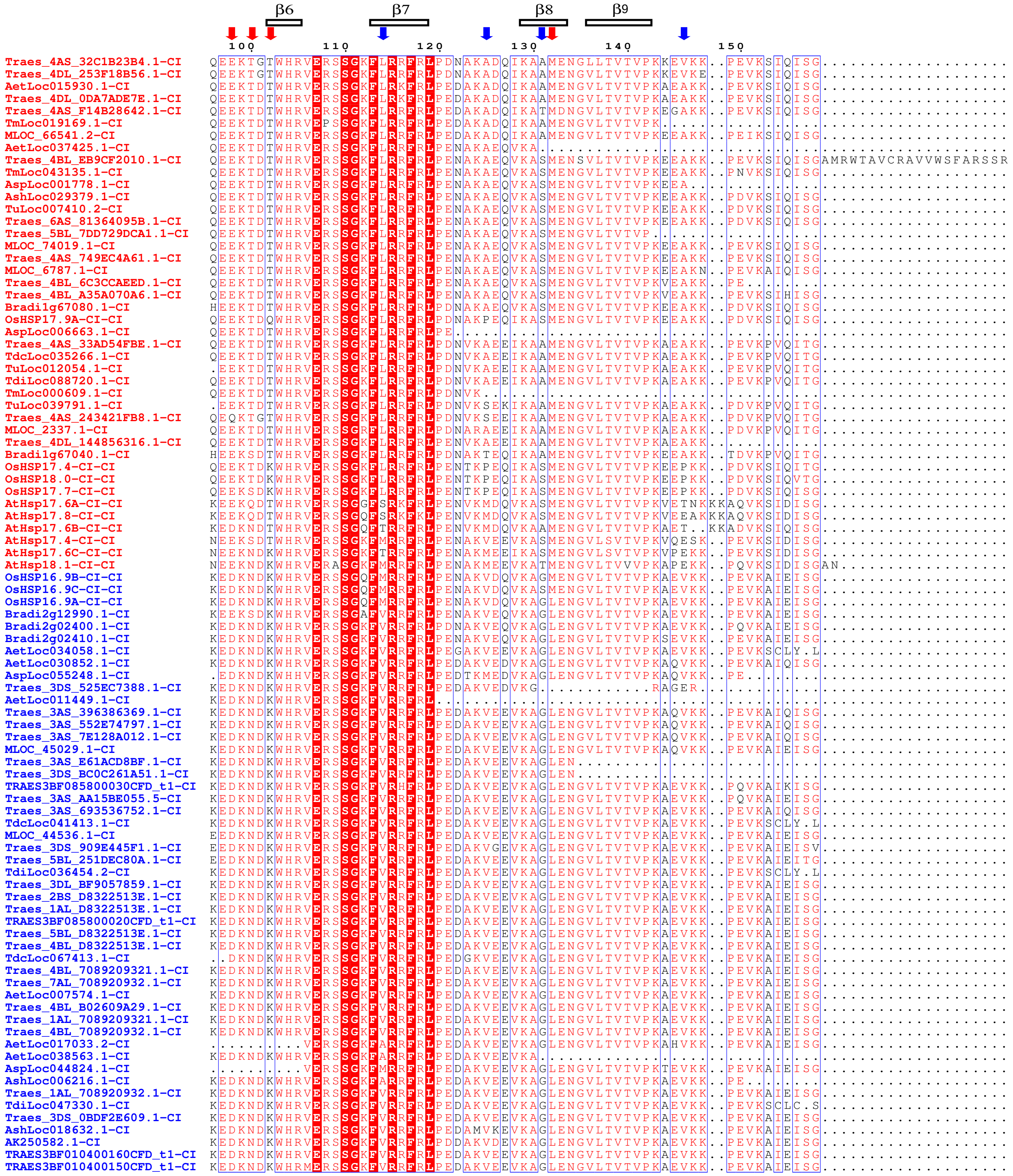

Figure a. Amino acid alignment of CI subfamily. The sequences were aligned to each other and to the cytosolic I protein of wheat sHSP *Ta*16.9 whose crystal structure was reported. The β strands were denoted by black rectangles on top. The columns which were colored in red and framed in blue had a greater than 70% similarity score, and the columns which were printed on red backgrounds were completely conserved in this subfamily. By phylogenetic tree, this subfamily could be divided into two groups: CIa (red sequences name) and CIb (blue sequences name), and the blue and red arrows marked sites that contribute to TypeI and TypeII functional divergence between two groups, respectively.

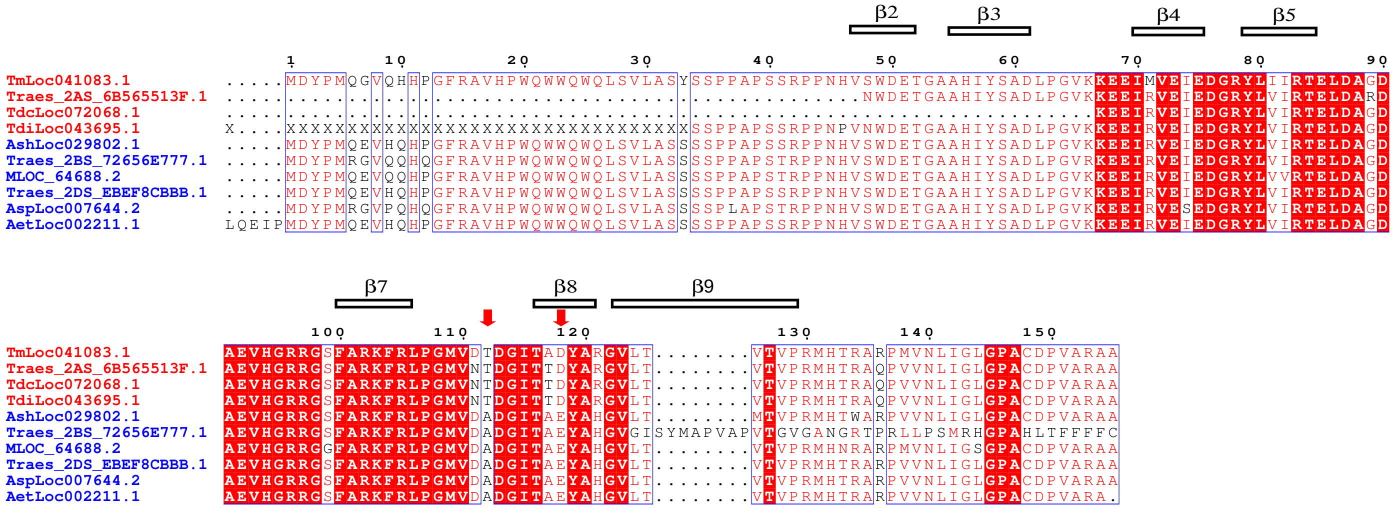

Figure b.Amino acid alignment of CV subfamily. The sequences were aligned to each other and to the cytosolic I protein of wheat sHSP *Ta*16.9 whose crystal structure was reported. The β strands were denoted by black rectangles on top. The columns which were colored in red and framed in blue had a greater than 70% similarity score, and the columns which were printed on red backgrounds were completely conserved in this subfamily. By phylogenetic tree, this subfamily could be divided into two groups: CVa (red sequences name) and CVb (blue sequences name), and the red arrows marked sites that contribute to TypeII functional divergence between two groups.

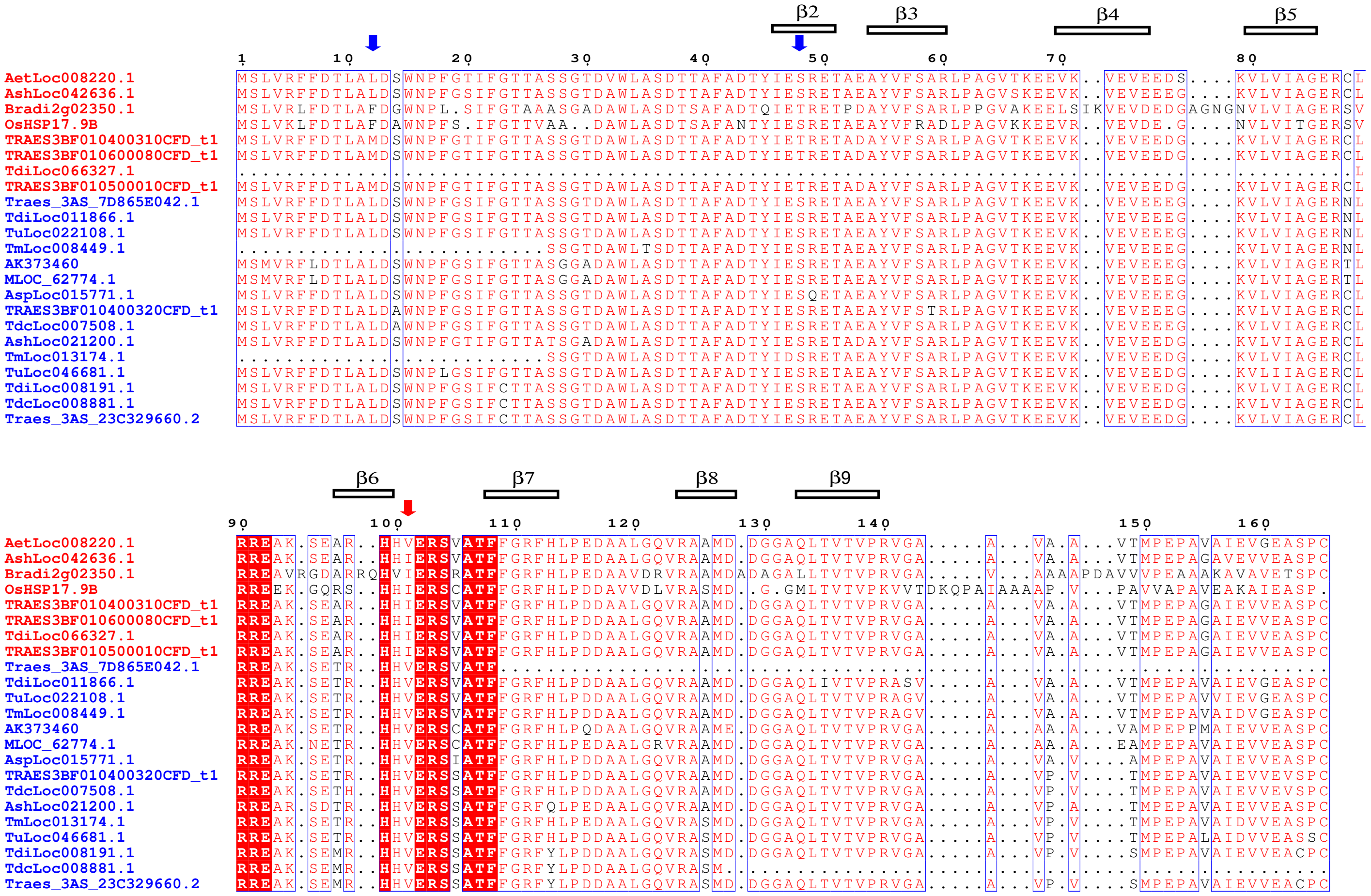

Figure c.Amino acid alignment of CIX subfamily. The sequences were aligned to each other and to the cytosolic I protein of wheat sHSP *Ta*16.9 whose crystal structure was reported. The β strands were denoted by black rectangles on top. The columns which were colored in red and framed in blue had a greater than 70% similarity score, and the columns which were printed on red backgrounds were completely conserved in this subfamily. By phylogenetic tree, this subfamily could be divided into two groups: CIXa (red sequences name) and CIXb (blue sequences name), and the blue and red arrows marked sites that contribute to TypeI and TypeII functional divergence between two groups, respectively.

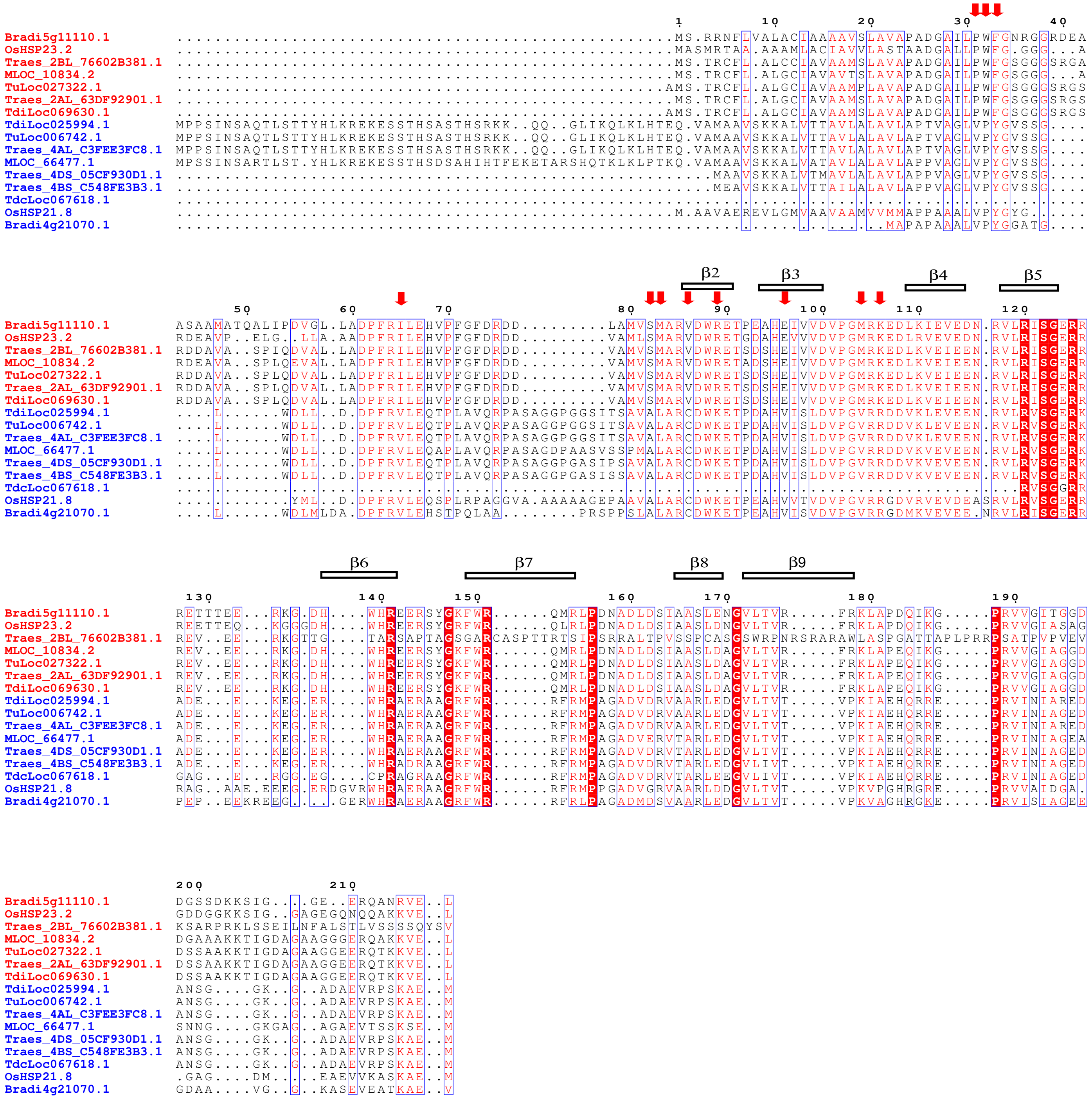

Figure d. Amino acid alignment ofER subfamily. The sequences were aligned to each other and to the cytosolic I protein of wheat sHSP *Ta*16.9 whose crystal structure was reported. The β strands were denoted by black rectangles on top. The columns which were colored in red and framed in blue had a greater than 70% similarity score, and the columns which were printed on red backgrounds were completely conserved in this subfamily. By phylogenetic tree, this subfamily could be divided into two groups: ERa (red sequences name) and ERb (blue sequences name), and the red arrows marked sites that contribute to TypeII functional divergence between two groups.

Traes_6DL_3BCB83718.2	....
TmLoc014962.2	....
AetLoc000977.1	....
AspLoc041930.2	....
AshLoc023559.2	....
Traes_6AL_904D2D9AB.1	....
TdcLoc022912.1	....
TuLoc024122.1	....
TdiLoc015137.2	....
MLOC_75175.1	MASRVVASSRIPLVRAMENLLAASSGP.....................ALRPAAVSVGVRGYNA.GAPLRSHD.RDEALEDTRHG.GRGA....R
Bradi3g58590.1	M.ASMAASKRVPLVRAMEKLLAAASAPG.................AGSALRPVAVAGGLRGYNT.GAQLRRYE.RDDSDDDVSR..........R
OsHSP24.0	M.ASIVASKRIPLFRLVEQLLAASPAQG.................AASALRPVAVAGGSRAYNT.GAQLRRHE.RDESDDDSGRGYDTRR....P
OsHSP26.2	M.ASTVALKGRPLATLLRQLLAADAPPAATGRPVAAAPAASGKPVTAPAAATATNAASRRLYNTEGAPLRRYDVVDESGTDS...GDEYDA..TD
AetLoc022522.1	....
Traes_7DS_58D823190.1	....
TdcLoc016988.1	....
TdiLoc049916.1	....
AetLoc015345.2	....M...MQEFQS.....NP.......I..L..RR
Traes_7DS_EEE208470.1	....
AshLoc009497.1	E.G.................RLY......................DDDDSSSS.........SS...GESSSE.....YE.....DTDSDD..RR
TdiLoc000063.1	....
AspLoc017241.2	....DDDS.........SS...GESGSE.....YE.....DTDSDD..RR
Traes_7AS_9E434596C.1	....
TuLoc030834.1	....
TmLoc013954.1	....F.................................YIV..G..AN
Traes_2BL_AB5025D19.1	M.R..R...CISPAT............TTTTT....T
TdiLoc035527.2	....
TdcLoc087152.2	....
AK249241.1	M.ASAVACKGAAPASLLMSGAPV......................AFCPHNTTAVIADRRPYNT...LVKEAI............RYDDD....H
AK372933	M.ASAVACKGATPASLLKSGAPV......................AFCPHNTTAVIADRRPYNT...LVKEAI............RYDDD....H
AK250452.1	M.ASAVACKDAAPASLLKSGAPV......................AFCPLSTTAITADRRPYNT...LIKEAI............RYDDDD....
AK370932	M.ASAVACKGATPASFLKSGAPV......................AFCPLNTTAVTADHRPYNT...LVKEAI............RYDDDD..DY
AetLoc014888.2	....
Traes_7DS_A7468F6DA.1	....
TmLoc016399.3	....PYST...QVKEVN............RYDDDD..DY
Traes_7AS_4B8075360.1	....
TuLoc034738.2	....ST...QVKEVN............RYDDDDDDDY
Traes_7DS_2A16E94E2.1	....
Traes_7BS_54E299396.1	....
Traes_7BS_261880A33.1	....
TdiLoc070274.1	....
AetLoc009818.1	....
Traes_7DS_FDFC9633D.1	....
Traes_7DS_00342ECC2.1	....
Traes_7BS_C222B0121.1	....
Traes_7BS_1E34E3CCC.1	....
Traes_7DS_7E6719BC2.1	....
Traes_5BL_E7B757C28.1	....
Traes_7DS_28AFF417D.1	....
TuLoc051037.1	....
TuLoc038248.2	M.ASAVACKGAAPVIPL..
Traes_7AS_5845260EC.1	....
TdiLoc089692.1	....
AshLoc020868.1	....
TmLoc018529.1	....
AetLoc029764.1	....
TmLoc018326.1	....
Traes_7DS_4E230B50B.1	....
Traes_7AS_E0F56CCBF1.2	....
TmLoc020830.1	....
AshLoc036635.1	....
TdiLoc074381.1	....
TuLoc028612.3	....
TmLoc018723.1	....
TuLoc011687.1	....
Traes_7BS_6C54E9123.1	....
TmLoc018191.1	....

```
                                                                                                β2         β3
                                               1                                  10         20        30        40
Traes_6DL_3BCB83718.2   .......................M....................LDDVS...A...ASPDGAARA.APMRRGWNAREDADALRLRVDMPGLGKEH
TmLoc014962.2           .......................CADVFRDPFSAPQSLGRLLSMLDDVA...A...ASPDGAARA.APLRRGWNAREDADALRLRVDMPGLGKEH
AetLoc000977.1          .......................RADVFRDPFSAPQSLGRLLSMLDDVS...A...ASPDGAARA.APMRRGWNAREDADALRLRVDMPGLGKEH
AspLoc041930.2          .......................CADVFRDPFSAPQSLGRLLSMLDDVA...A...ASPDGAARA.APMRRGWNAKEDADALRLRVDMPGLGKEH
AshLoc023559.2          .......................VQHVFRDPFSAPQSLGRLLSMLDDVA...A...ASPDGAARA.APLRRGWNAKEDADALRLRVDMPGLGKEH
Traes_6AL_904D2D9AB.1   ............................................................................................KEH
TdcLoc022912.1          ....................................................................MRRGWNAREDADALRLRVDMPGLGKEH
TuLoc024122.1           .......................CADVFRDPFSAPQSLGRLLSMLDDVA...A...ASPDGAARA.APLRRGWNAREDADALRLRVDMPGLGKEH
TdiLoc015137.2          .......................CADVFRDPFSAPQSLGRLLSMLDDVA...A...ASTDGAARA.APLRRGWNAREDADALRLRVDMPGLGKEH
MLOC_75175.1            ERG.......ASVPS.F......FSDVFRDPFSAPQSLGRLLNMMDDVA...A...AGAPVGARA.APLRLGWNAKEDEDALRLRVDMPGLGKEH
Bradi3g58590.1          DAR.......NVAVP.S.....FFSDVFRDPFSSPQSLGRLLSLMDDMA...A...PGG....RA.ATVRRGWNAKEDEEALHLRVDMPGLGKEH
OsHSP24.0               TRD.......ATMPA.F.....FSGNVFRDPFSAPQSLGRLLSLMDDLA...T...PAG..RAGA.ATLRRGWNAKESEEALHLRVDMPGLGKEH
OsHSP26.2               DGR.......RLTVPFF.....FSASDVLDPFGAPTSLGRLLALMEDAAVATAAAPGTNGL.ATAAARRGGWWVAKEDDDAVHLKVSMPGLGKEH
AetLoc022522.1          .......................................................................................MLGLGKEC
Traes_7DS_58D823190.1   ............................................MEDGA...A...PPGLS.SAAGAPRRGWWVEKDDDDAVYLTVPMLGLGKER
TdcLoc016988.1          ...............................................................................................
TdiLoc049916.1          ...............................................................................................
AetLoc015345.2          ATL.......SVGAP.A.....ESLGRRAGQFGAHTSLGRLLSALEDAA...A...PTGLS.STAGASRLGRWVAKVDDDAVYLKVPMPGLGKEH
Traes_7DS_EEE208470.1   ...............................................................................................
AshLoc009497.1          RARDFFVPSFSQAFL.V.....LDSPDVLDQFGAHTSLGRLLSALEDAA...A...PTGLS.STAGASRLGRWVAKVDDDAVYLKVPMPGLGKEH
TdiLoc000063.1          ..................................................................ASRLGRWVSKVDDDAVYLKVPMPGLGKEH
AspLoc017241.2          RARDFFVPSFSQA..............................ALEDVA...A...PTGLS.STAGASRLGRWVSKVDDDAVYLNVPMPGLGKEH
Traes_7AS_9E434596C.1   ...............................................................................................
TuLoc030834.1           ...................................................................................LKVPMPGLGKEH
TmLoc013954.1           R.GS......DQLFL.V.....LDSPDVLDQFGAHTSFGRLLSVLEDAA...A...PTGLS.STAGASRLGRWVSKVDDDAVYLKVPMPGLGKEH
Traes_2BL_AB5025D19.1   AAV.......TSSSP.ASSCRDVDSADMLEPHNAPTSKARLLSLMET.........G..LS.STVGMSRLGRWLTKEDNDAVYLKVPMSGMTKEQ
TdiLoc035527.2          ............VIP.S.....FFLQDMLEPHNAPTSKARLLSLMET.........G..LS.STVGMSRLGRWLTKEDNDAVYLKVPMSGMTKEQ
TdcLoc087152.2          ............VIP.S.....FFLQDMLEPHNAPTSKARLLSLMET.........G..LS.STVGMSRLRRWLTKEDNDAVYLKVPMSGMTKEQ
AK249241.1              SGR.......DLVIP.S.....FISQDVLDPLGAPTSMARLLSLMEDVS...T...QTGLS.STAGASRLGRWVAKEDDGAVYLKVPMPGLTKEH
AK372933                SGR.......DLVIP.S.....FISQDVLDPLGAPTSMARLLSLMEDVS...T...QTGLS.STAGASRLGRWVAKEDDGAVYLKVPMPGLTKEH
AK250452.1              D.R.......NLVLP.S.....FFSQDVLDPLGAPTGIARLLSQ..........T...GLS.STAGASRLGRWVAKEDDGAVYLKVPMPGLTKEH
AK370932                SGR.......HLVLP.S.....FFSQDVLDPLGAHQ.....................TGLS.CTAGASRLGRWVTKEDDGAVYLKVPMPGLTKEH
AetLoc014888.2          ............AAPAA.....LLKSDVLDPLGAPTGMARLLSLMEDVA...T...QTGLS.TTAGASRLGRWVAKEDDDAVYLKVPMPGLTKEH
Traes_7DS_A7468F6DA.1   .......................................................................................MPGLTKEH
TmLoc016399.3           SDR.......DLVIP.S.....SFSQ................ALMEDVA...A...QTG.L.STAGASRLGRWVAKEDDGAVYLKVPMPGLTKEH
Traes_7AS_4B8075360.1   ...............................................................................................
TuLoc034738.2           SDR.......DLVIP.S.....SFSQ................ALMEDVA...A...QTG.L.STAGASRLGRWVAKEDDDAVYLKVPMPGLTKEH
Traes_7DS_2A16E94E2.1   .......................................................................................MPGLTKEH
Traes_7BS_54E299396.1   .......................................................................................MPGLTKEH
Traes_7BS_261880A33.1   ...........MARL.L.....SLME.................DVATKT......GGLSST.AGAGASRLGRWVAKEDDDAVYLKVPMPGLTKEH
TdiLoc070274.1          ...........................................................................................TKEH
AetLoc009818.1          ...............................................................................................
Traes_7DS_FDFC9633D.1   .......................................................................................MPGLTKEH
Traes_7DS_00342ECC2.1   .......................................................................................MPGLTKEH
Traes_7BS_C222B0121.1   .......................................................................................MPGLTKEH
Traes_7BS_1E34E3CCC.1   .......................................................................................MPGLTKEH
Traes_7DS_7E6719BC2.1   .......................................................................................MPGLTKEH
Traes_5BL_E7B757C28.1   .......................................................................................MPGLTKEH
Traes_7DS_28AFF417D.1   ...............................................................................................
TuLoc051037.1           ..........................................................SST.AGAGASRLGRWVAKEDDDAVYLKVPMPGLTKEH
TuLoc038248.2           ........................KSDVIDPLSAPTSIARLLSLMEDVA...TRTGGLSSI.AGAGASRLRRWVAKEDDDTVYLKVPMPGLTKEH
Traes_7AS_5845260EC.1   ...............................................................................................
TdiLoc089692.1          .........................................................................VAKEDDDAVYLKVPMPGLTKEH
AshLoc020868.1          ...............................................................................................
TmLoc018529.1           ...................................................................................LKVPMPGLTKEH
AetLoc029764.1          ...............................................................................................
TmLoc018326.1           .........................................................................................GLTKEH
Traes_7DS_4E230B50B.1   .......................................................................................MPGLTKDH
Traes_7AS_E0F56CCBF1.2  ...............................................................................................
TmLoc020830.1           .........................................................................................GLTKEH
AshLoc036635.1          ...............................................................................................
TdiLoc074381.1          .....................................................GG..GDS..TANGGLSPGRWILSCVPRAWALGARGPAPPLDH
TuLoc028612.3           ..........DLVTP.S.....FFSQ................DVLDSLG...A..GGLSST.AGAGASQLGRWVAKEDDDAVYLKVPMPGLTKDH
TmLoc018723.1           ...............................................................................................
TuLoc011687.1           ...............................................................................................
Traes_7BS_6C54E9123.1   .......................................................................................MPGLTKEH
TmLoc018191.1           ...............................................................................................
```

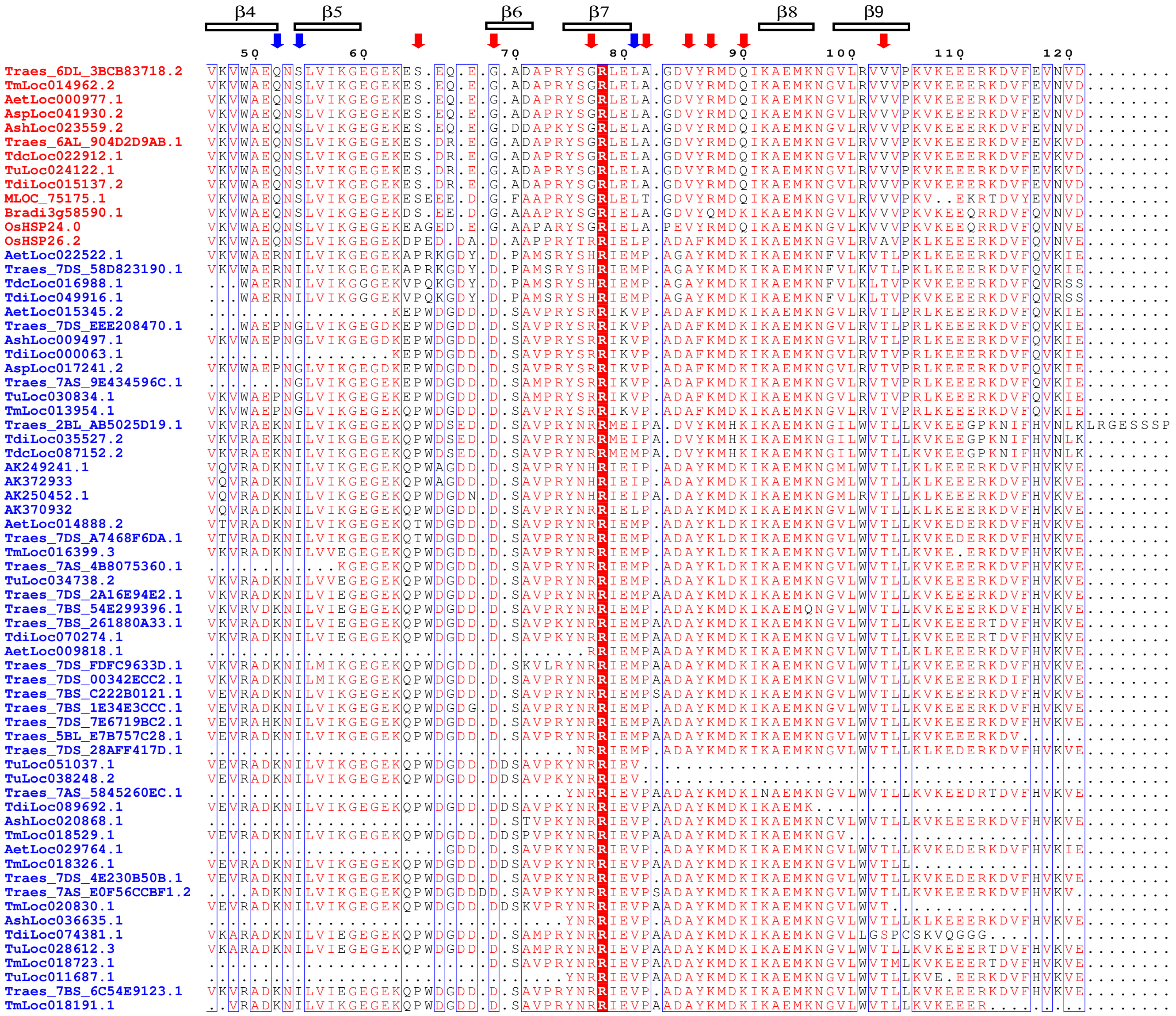

Figure e. Amino acid alignment of MI subfamily. The sequences were aligned to each other and to the cytosolic I protein of wheat sHSP *Ta*16.9 whose crystal structure was reported. The β strands were denoted by black rectangles on top. The columns which were colored in red and framed in blue had a greater than 70% similarity score, and the columns which were printed on red backgrounds were completely conserved in this subfamily. By phylogenetic tree, this subfamily could be divided into two groups: MIa (red sequences name) and MIb (blue sequences name), and the blue and red arrows marked sites that contribute to TypeI and TypeII functional divergence between two groups, respectively.

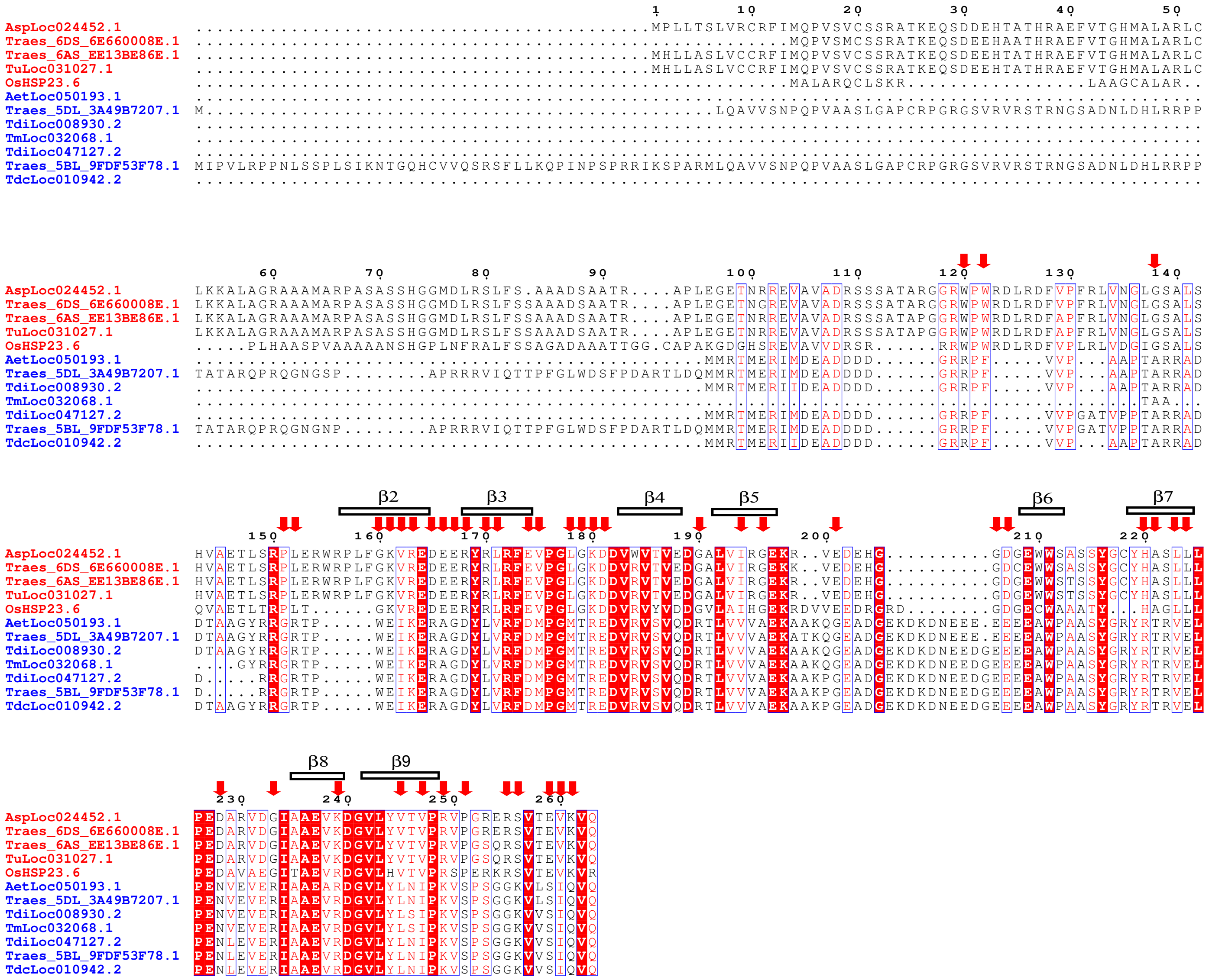

Figure f. Amino acid alignment of MII subfamily. The sequences were aligned to each other and to the cytosolic I protein of wheat sHSP *Ta*16.9 whose crystal structure was reported. The β strands were denoted by black rectangles on top. The columns which were colored in red and framed in blue had a greater than 70% similarity score, and the columns which were printed on red backgrounds were completely conserved in this subfamily. By phylogenetic tree, this subfamily could be divided into two groups: MIIa (red sequences name) and MIIb (blue sequences name), and the red arrows marked sites that contribute to TypeII functional divergence between two groups.

Supplementary Figure S6

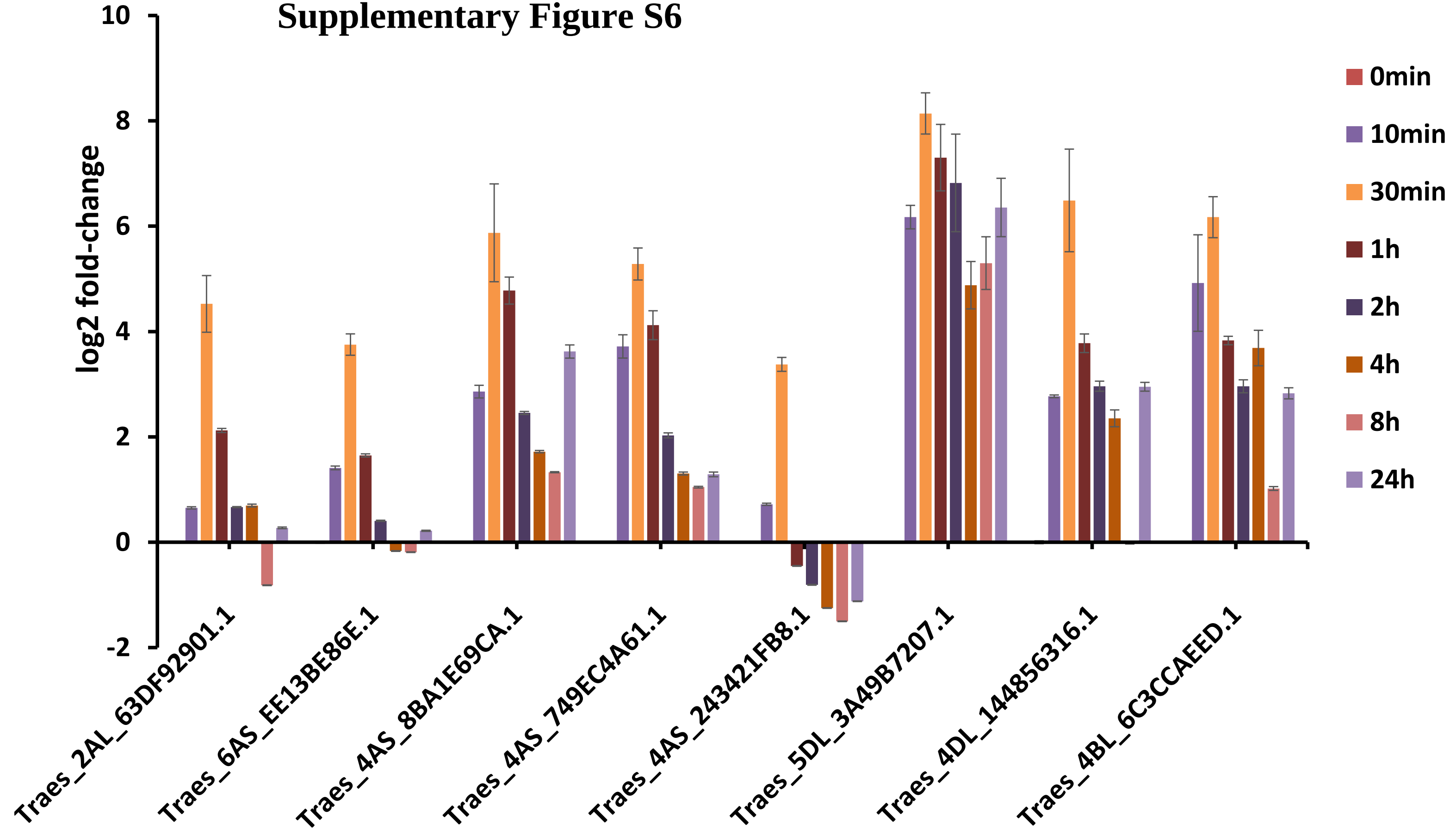

Supplementary Table S1. SHSPs numbers of each subfamily in different species.

	At	Os	Bd	Hv	AA^{mm}	AA^{uu}	$S^{sh}S^{sh}$	SS	DD	$AABB^{Tdc}$	$AABB^{Tdi}$	Ta^{AA}	Ta^{BB}	Ta^{DD}	Ta
Acd	9	12	11	11	8	6	10	11	9	10	12	7	6	4	17
CI	6	7	6	9	6	5	8	6	10	7	6	18	17	11	46
CII	2	2	1	4	0	2	3	1	3	1	4	3	9	4	16
CIII	1	1	1	1	1	3	5	5	2	2	6	1	1	1	3
CIV	1	0	0	0	0	0	0	0	0	0	0	0	0	0	0
CV	1	1	1	1	1	0	1	1	1	1	1	1	1	1	3
CVI	1	1	1	1	0	1	0	0	0	0	0	0	0	0	0
CVII	1	0	0	0	0	0	0	0	0	0	0	0	0	0	0
CVIII	0	1	0	0	0	0	1	1	2	1	1	0	1	0	1
CIX	0	1	1	2	2	2	2	1	1	2	3	2	4	0	6
CX	0	1	1	1	1	1	1	1	1	1	2	1	0	0	1
CXI	0	1	0	0	0	0	0	0	0	0	0	0	0	0	0
CXII	0	0	0	0	1	1	1	1	1	2	2	0	1	0	1
ER	1	2	2	2	0	2	0	0	0	1	2	2	2	1	5
MI	2	2	1	5	9	8	7	4	8	4	9	6	9	10	25
MII	1	1	0	0	1	1	0	1	1	1	2	1	1	2	4
P	1	1	1	1	2	2	0	2	0	1	0	3	1	1	5
Px	1	1	1	1	1	1	1	1	1	1	1	1	0	0	1
total	19	23	17	28	25	29	30	25	31	25	39	39	47	31	117

Abbreviations: At, *Arabidopsis thaliana*; Os, *Oryza sativa*; Bd, *Brachypodium distachyon*; Hv, *Hordeum vulgare*; AA^{mm}, *Triticum monococcum*; AA^{uu}, *Triticum urartu*; $S^{sh}S^{sh}$, *Aegilops*. *Sharonensis*; SS, *Aegilops speltoides*; DD, *Aegilops tauschii*; $AABB^{Tdc}$, *Triticum durum*, cv. Strongfield; $AABB^{Tdi}$, *Triticum durum*, cv. Cappelli; Ta^{AA}, subgenome AA of bread wheat; Ta^{BB}, subgenome BB of bread wheat; Ta^{DD}, subgenome D of bread wheat; Ta, *Triticum aestivum*.

Supplementary Table S2. Estimates of codon-substitution evolutionary models for different groups in CI, CV, CIX, ER, MI and MII subfamilies.

Comparison	Model		Ln L	*P* value
Branch leading to CIa vs. CIb	Model 0		-11163.642798	0.12
	Two ratio Model 2		-11162.462948	
	Model 3		-10574.641869	9.84E-009
	Model D		-10558.205925	
	Branch leading to CIa	Model A null	-10740.792810	0.04
		Model A	-10738.782751	
	Branch leading to CIb	Model A null	-10740.791960	0.04
		Model A	-10738.775244	
Branch leading to CVa vs. CVb	Model 0		-1291.279277	NA
	Two ratio Model 2		-1292.174241	
	Model 3		-1272.768934	4.5E-004
	Model D		-1266.613408	
	Branch leading to CVa	Model A null	-1273.556302	0.39
		Model A	-1273.187843	
	Branch leading to CVb	Model A null	-1273.556338	0.37
		Model A	-1273.161686	
Branch leading to CIXa vs. CIXb	Model 0		-2186.645681	7.08E-003
	Two ratio Model 2		-2183.019705	
	Model 3		-2153.209652	0.22
	Model D		-2152.461463	
	Branch leading to CIXa	Model A null	-2165.776869	1
		Model A	-2165.776869	
	Branch leading to CIXb	Model A null	-2165.776869	0.99
		Model A	-2165.776938	
Branch leading to ERa vs. ERb	Model 0		-4884.968044	1.85E-006
	Two ratio Model 2		-4873.593374	
	Model 3		-4812.917631	1.52E-002
	Model D		-4809.969598	
	Branch leading to ERa	Model A null	-4845.966316	3.56E-003
		Model A	-4841.719011	
	Branch leading to ERb	Model A null	-4845.966323	3.39E-003
		Model A	-4841.674451	
Branch leading to MIa vs. MIb	Model 0		-7237.896335	1.66E-002
	Two ratio Model 2		-7235.026267	
	Model 3		-6840.068035	3.44E-003
	Model D		-6835.787937	
	Branch leading to MIa	Model A null	-6868.046568	0.28
		Model A	-6867.468571	
	Branch leading to MIb	Model A null	-6868.046573	0.28

		Model A	-6867.465253	
Branch leading to MIIa vs. MIIb	Model 0		-3571.068441	0.61
	Two ratio Model 2		-3570.941784	
	Model 3		-3534.352386	1.34E-002
	Model D		-3531.294731	
	Branch leading to MIIa	Model A null	-3548.332879	NA
		Model A	-3561.677418	
	Branch leading to MIIb	Model A null	-3548.332879	NA
		Model A	-3554.142067	

Asymmetric evolution detection: One ratio Model 0 vs. Two ratio Model 2.

Detection of selective pressure among paralogous clades: Model 3 vs Model D.

Detection of positively selected sites: Model A null (ω is fixed as 1) vs. Model A.

Degree of freedom (df) = 1 for all the comparisons. For clade names of each subfamily, see additional file Figure S3 legend.

Supplementary Table S3. Statistical test of expression patterns between duplicated groups in CI, ER, MI and MII subfamilies.

**Duplicated groups**	**F test**	**T test**
**CIaa**	1.93486E-14	0.000468747
**CIab**		
**CIba**	7.3618E-192	2.05604E-08
**CIbb**		
**ERa**	2.14287E-57	0.015982446
**ERb**		
**MIa**	6.2814E-117	0.000140482
**MIb**		
**MIIa**	3.06665E-15	0.012062436
**MIIb**		

Supplementary Table S4. The primers unsed for Real time PCR.

Gene	Forward primer	Reverse primer
Traes_4BL_6C3CCAEED.1	CCCCTTCTCCCTCGACCTCT	ACGGGCGTCTCCTTCCA
Traes_4AS_749EC4A61.1	CCCCTTCTCCCTCGACTTCT	TCGGGCGTCTCCTTCCA
Traes_4DL_144856316.1	CATCTTCCCACGCACCACC	TCCACCTCAACCTTCACCTCC
Traes_4AS_243421FB8.1	CCCCTTCTCGCTCGACCTCT	CCTCGGGCATCTCCTTCCA
Traes_2AL_63DF92901.1	TAAAGGTGGAGATCGAGGAGAACC	CGCCAGAACTTGCCGTAGGA
Traes_6AS_EE13BE86E.1	GAGCGACCAAAGAGCAAAGTG	CCGTGAGAACTGGCTGATGC
Traes_4AS_8BA1E69CA.1	GGTGAGGGTGATGGTGGAGG	CAGCACGCCGTTCTTGAGC
Traes_5DL_3A49B7207.1	GACTACCTGGTGCGGTTCGA	CCACGCCTCCTCTTCCTCCT
Actin	CTCCCTCACAACAACCGC	TACCAGGAACTTCCATACCAAC
